# Dynamics of internal migration in Bangladesh: Trends, patterns, determinants, and causes

**DOI:** 10.1371/journal.pone.0263878

**Published:** 2022-02-14

**Authors:** Md. Zakiul Alam, Abdullah Al Mamun

**Affiliations:** Department of Population Sciences, University of Dhaka, Dhaka, Bangladesh; Shahjalal University of Science and Technology, BANGLADESH

## Abstract

**Introduction:**

Internal migration is essential to understand the population dynamics and the multifaceted relationship between population and development of a nation. In Bangladesh, the study of international migration is more frequent due to its socioeconomic importance and data availability. However, the study of internal migration is less frequent as there lie complexities in measuring internal migration, and data are less available. Thus, this paper aimed to explore the dynamics of internal in Bangladesh.

**Data and methods:**

We utilized data from the Bangladesh Population and Housing Census 1991–2011. The number of internal migrants was estimated using the United Nations Manual on *Methods of Measuring Internal Migration- Manual VI*. District-wise lifetime and net internal migration rate were the dependent variables where several socioeconomic variables were used as independent variables. The correlation and the stepwise multiple linear regression analysis were employed.

**Results:**

Dhaka, Gazipur, Narayanganj, and all the Divisional cities have the highest in-migration rate, whereas the northern and southern districts of Bangladesh have the highest out-migration rate. The regression model showed that activity rate appeared to be the strongest predictor (β = 0.419, P<0.001) of net migration for 2011, followed by city corporation (β = 0.275, P<0.01) and poverty rate (β = -0.246, P<0.01). However, the lifetime internal migration rate was 9.8% in 2011. The pooled model (1991–2011) for lifetime internal migration showed that activity rate (β = 0.408, P<0.001), population density (β = 0.386, P<0.001), literacy rate (β = 0.341, P<0.001), and city corporation (β = 0.139, P<0.01) were the significant factors of internal migration. Marriage, looking for a job, employment/business, education, and natural calamities were the reasons for internal migration.

**Discussion and conclusion:**

The destinations of migrants are few developed and urbanized cities which needs particular attention in policy planning. If the current migration trends continue, few cities will have an excessive population, which will increase density and pollution, thereby decreasing living standards. Thus, along with comprehensive urban planning, decentralization of government and private institutions must be ensured. Since the rural to urban migration rate is high, the findings recommend more development and concentration in the rural area. Finally, education, training, and work opportunities for migrants should be safeguarded in the area of origin.

## Introduction

As the fundamental component of population change, migration is generally the movement of people from one geographic location to another to live permanently or temporarily -. Various countries worldwide are experiencing migration as a significant component in shaping population distribution between and within countries after completing the first demographic transition [1]. As a complex phenomenon in demography, migration is rarely analyzed and discovered in a third-world country, where data are primarily incomplete, and their quality is questionable [1, 2]. The study of international migration is more frequent as its data are more available than internal migration, and many organizations monitor the international migration sector in Bangladesh [3–6]. Nevertheless, two-thirds of the migration in Bangladesh is internal [7], which has greater importance as a factor of people’s livelihood and shapes the country’s economy and development [7–9]. Therefore, the study of internal migration is significant in Bangladesh for understanding population distribution, growth, and urbanization [8, 10].

Internal migration has generally been considered the significant urbanization process [11], becoming a foremost policy concern in Bangladesh. After the independence in 1971, the urbanization rate of Bangladesh has been more than 3% [12], and it is anticipated that the urban population will increase day by day. The final stage of demographic transition (youth bulge), accelerated economic transformation due to structural shift (agriculture to industry and services), and climate change are the foremost reasons behind the unprecedented internal migration in Bangladesh. Since there are no administrative restrictions, the extent of unplanned urbanization is increasing and causing many slums in Bangladesh, which pose excessive pressure on the environment, health, and economy [5, 13, 14]. Thus, internal migration has often been treated as both hero and villain of national development [8].

In contrast, the influence of international migration on the economy has mainly been positive [15], where 3.3% of migrants of the world population contributed 9.4% of global GDP [6, 16]. Bangladesh is one of the largest countries of origin, where the share of remittances approaches 10% of GDP [8]. As a result, many studies have been conducted on international migration. However, internal migration is less discovered in Bangladesh because it does not affect population change but population distribution. Nevertheless, there is a positive relationship between internal migration and the economy, where internal migration is considered a rational decision to move out from poverty [2, 17]. Rural to urban migration areas have been adopted as a livelihood strategy by many families who migrate for better employment opportunities in Bangladesh [9]. This migration is an adjustment instrument that changes underemployed and unemployed from the local labor market to areas that can be fully employed through equilibrium. Existing studies also show that socioeconomic factors, conflicts, and climatic factors are the foremost factors of internal migration [9, 14, 18–21]. However, migration not only occurs from rural to urban but also from urban to rural [10], which is a distinctive feature of migration.

The first influential work of internal migration in Bangladesh was in 1992, where the author tried to show the trends and determinants of internal migration using census data between 1974 and 1981 [2]. Then, there have been found a substantial number of works [4, 7–9, 13, 14, 19, 22–24]. However, most of the works are either of the small-scale survey [7, 9, 14, 19, 22, 23], which cannot be generalized for the whole country or used the only lifetime internal migration data collected asking place of birth from the respondents [8, 10]. The use of both lifetime internal migration (as direct method) and estimated internal migration with the help of UN Manual VI (as an indirect method) [25, 26] would be more comprehensive and generalizable. In this regard, we aimed to analyze the trends, patterns, determinants, and causes of internal migration using the census data of Bangladesh from 1991 to 2011 using both direct and indirect methods. The findings of this study will help to initiate policy and program planning regarding population redistribution in Bangladesh.

### Conceptual framework of internal migration

Migration affects and is affected by numerous socioeconomic, demographic, political, and environmental factors. Therefore, the development of universal migration theory is hindered due to the dynamic and complex nature of migration [27, 28]. Several theories have been developed since the first effort of Ravenstein (laws of migration) from the distinct perspectives of sociology, economics, political science, geography, and related disciplines. These theories are broadly two types: a) functionalist b) historical-structural [27]. Functionalist theories of migration such as the Push-pull model, neo-classical equilibrium model, migration systems theory, the new economics of migration state that migration is a rational decision of individual or family based on cost-benefit analysis [27–29]. The Push-pull theory proposed by Everett Lee in 1966 discussed that the push factors are related to the area of origin, whereas pull factors are linked with the area of destination [30]. The push-pull factors chase people away from an area and attract them to a new location. Combining push-pull factors determines the emigration or immigration of particular populations from one place to another. This theory can be thought of as a prototype of the neoclassical migration model [27]. The neoclassical model discusses migration from the economic premise and states it as a process of labor adjustment and human capital between the area of origin and destination due to socioeconomic differences [31]. Conversely, the new economics of migration theory proposed that family rather than individual decides to migrate to minimize family’s risk in poverty [32]. All these theories describe migration from a micro perspective [29].

Historical-structural theories of migration such as neo-Marxist conflict theory, world-systems theory, dual labor market theory, on the other hand, interpreted migration as a result of structural economic, and power inequalities; thus, they see migration from a macro perspective [29, 33–36]. Both types of theory have contributed to understanding the migration phenomena. The functionalist theories are criticized for their static nature (push-pull model) and inability to explain the social and geographical differentiated nature of migration. However, these theories are prominent in explaining internal migration from the micro-level [27]. On the other hand, the macro-level theory links internal migration with development, a socioeconomic structure that influences the decision at the micro-level. For example, Zelinsky’s mobility transition hypothesis associates the level and direction of migration with the demographic transition. The mobility transition hypothesis stated that early transitional society experiences large-scale rural to urban migration. In contrast, advanced and super-advanced society experiences a decline in rural to urban migration, and consequently, urban to urban migration increases [37]. Thus, all this complexity and contextual variation lead to the multilevel theory to understand internal migration [29].

**Fig 1:**
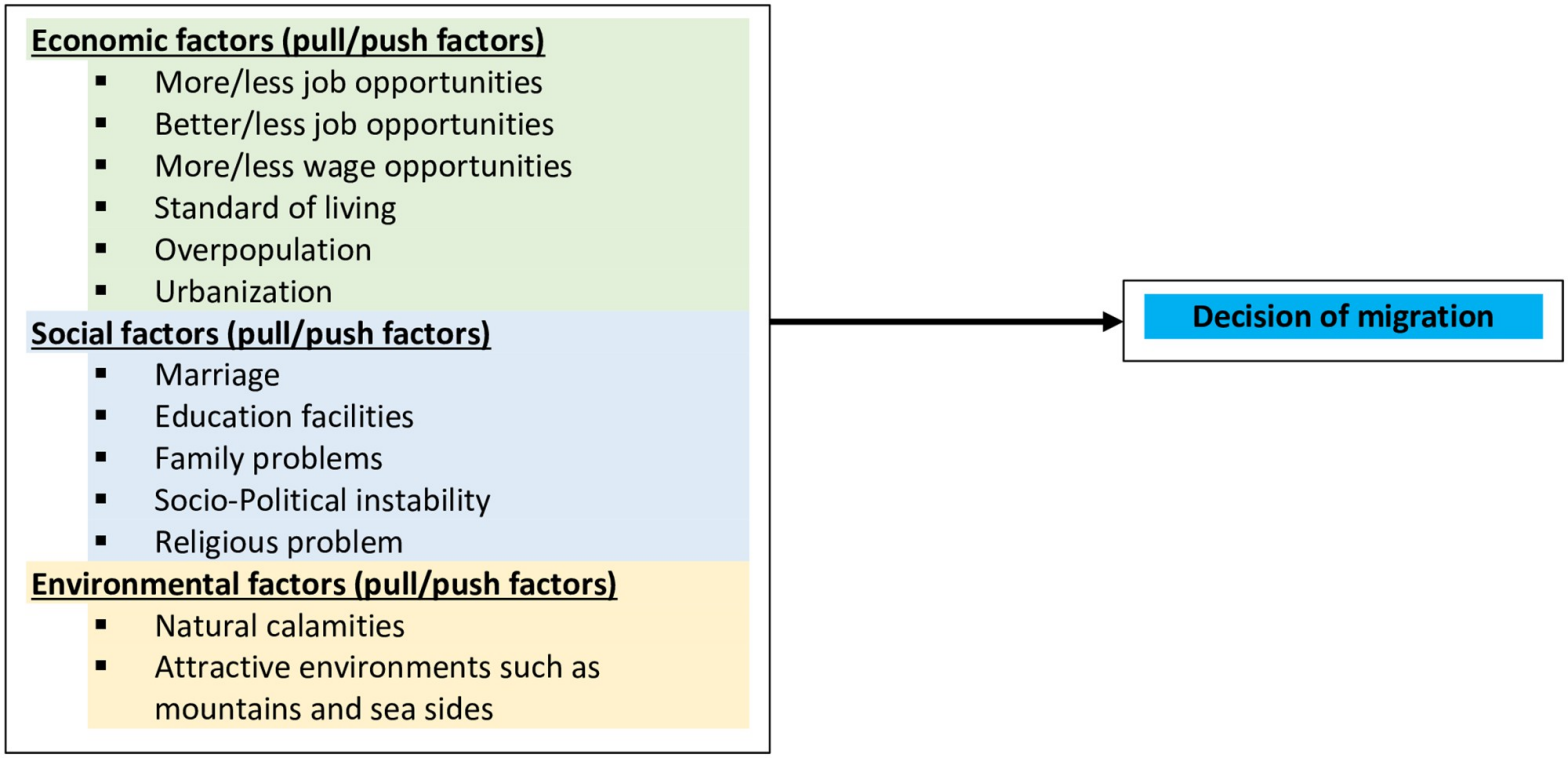
Conceptual framework of internal migration in Bangladesh. Source: The framework was produced using existing literature [2, 4, 7–11, 14, 18, 19, 22, 30, 31, 38].

Based on the above discussions, the causes of migration can broadly be categorized as economic, social, and environmental factors (Fig 1). Employability and business opportunities in the destination area are the vital economic premises that attract people for the movement [31]. On the other hand, less opportunity in the area of origin acts as the push factor. Similarly, social reasons tend to involve both voluntary and forced migration. In Bangladesh, marriage is a major cause of internal migration for women [10]. Better education facilities in the destination area also push the young population for migration. Family, political, and religious problems may also push the people to migrate. Finally, environmental factors like natural calamities work as a push factor [10].

## Data and methods

### Data sources

We used the Population and Housing Census data to analyze the dynamics of internal migration in Bangladesh from 1991 to 2011 [39–43]. Since the previous work used data from 1974 to 1981 [2], we used the between 1991 to 2011. The Census in the Indian subcontinent (Bangladesh as a part of it) was initiated in 1872, followed by 1881. Then, it was conducted with the decennial cyclicity and continued excluding 1971 because of the liberation war. The first-ever Census in Bangladesh was in 1974 after the rise as a new sovereign country in 1971. Bangladesh went back to the decennial periodicity and conducted the second, third, fourth, and fifth censuses in 1981, 1991, 2001, and 2011. However, the detailed methodology is available elsewhere in the report [39–43].

### Measures

#### Dependent variables

The dependent variables of this study were ‘lifetime internal migration’ (direct method) and ’net internal migration’ (indirect method) for each district. The questionnaire of the Census of Bangladesh had questions regarding birthplace and current living place, which was used to calculate lifetime internal migration [39–43] as a direct method [26]. However, these statistics are of limited use because they do not reflect the timing of the movement or how many times they moved [44].

For this reason, we estimated the number of net migrants for each district (an indirect method) using the United Nations’ Manual on Methods of Measuring Internal Migration- Manual VI [25, 26] to overcome the shortcoming of lifetime internal migration and make the analysis credible and comparable. Indirect estimation of internal migration can be done using data from sample vital statistics and Census. Methods based on vital statistics need registered data on birth and death for each district or region, which is not available in the context of Bangladesh. We had to use the “Census survival ratio” (CSR) method in this regard. We first estimated the CSR for each group. Then using the CSR, the expected population for each district/ region is calculated. The difference between expected and observed population by Census is attributed as net migrants using the following equation.


$$M_{i}(x)=P_{i,x+n,t+n}-\frac{P_{x+n,t+n}}{P_{x,t}}\times P_{i,x,t}$$

Here, M_i_ (x) is the number of internal migrants in the ’i’ districts and ’x’ age category and P_i, x+n, t+n,_ meaning population in ’i’ districts in ’x+n’ age category in ’t+n’ year. The causes of internal migration were also taken from the Census. Under this manual, the definition of internal migration is "the change of usual place of residence from one civil division (for this paper District) to another within the country" [25]. This procedure tends to correct for systematic errors in the age data (such as under enumeration of specific age groups) and compensate for the effect of such errors (Manual VI). In addition, the relative change of migration was measured using the difference between net migration of 2001 (between 1991 and 2001) and 2011 (between 2001 and 2011) divided by the 2001 migrants.

#### Independent variables

Data of independent variables were collected from the Analytical Report of Population and Housing Census of 1991, 2001, and 2011 [39–43]. All the data are presented in the annexed table (S1–S3 Tables). Here, the urban population (UR) represents the total population in a district living in an urban area. The activity rate (AR), used as a refined activity rate, is an economically active population of 10+ years as defined in the Analytical Report of Population and Housing Census. Literacy rate (LR) is defined as the ratio of literate (can write a letter in any language) population of 7+ years and total population. Population density per square kilometer (PD) holds the definition of the number of persons living per square kilometer. The never-married population is the ratio between the never married (male and female) population and the total population of 10+ years expressed in percentage. Average household size (HS) is the mean number of household members. The average HS was measured by dividing the entire population by the total household number for each district. The Division is the first-level administrative region in Bangladesh. There are currently eight divisions in Bangladesh, and each is named after the principal city within its jurisdiction. City corporation (CC) is some urban area incorporated and administered by the Local Government Division under the City Corporation Act 2009. There are 12 city corporations in Bangladesh. Divisional headquarters are part of CC. Finally, the poverty rate (PR) is the headcount ratio for each district, was used as an indicator of poverty only for 2011, taken from HIES 2010 [45].

### Analytical approach

We produced two maps (Fig 3) for net internal migration rate (indirectly estimated following the UN Manual VI) using ArcGIS: one for 2011 (migration rate between 2001 and 2011) and another for 2001 (migration rate between 1991 and 2001). Age-specific migration rates were presented using indirect estimation (Table 1). Correlation (Pearson and Spearman) was used as the bivariate analysis. Correlations were performed for only net internal migration (indirect) data (Table 3). We used stepwise multiple linear regression models to identify the socioeconomic predictors of internal migration in Bangladesh. The normality and homoscedasticity of the data were checked using the P-P normality plot of residuals and scatter plots. Due to multicollinearity, we dropped the urbanization rate and the proportion of the never-married population from multiple analyses. Regression models were separately produced for the lifetime and net internal migration.

**Table 1:** Net migration rate by district between 2001 and 2011.

District	5 Years Age Group													Overall
	10–14	15–19	20–24	25–29	30–34	35–39	40–44	45–49	50–54	55–59	60–64	65–69	70+	
Barguna	-0.6	-23.4	-34.4	-8.9	-0.3	-2.0	-10.3	-1.3	1.0	0.6	-5.5	-3.7	-10.3	-9.0
Barishal	-4.5	-23.0	-54.7	-35.7	-7.6	-5.2	-12.6	-9.0	-6.9	-13.5	-4.5	-4.7	-11.3	-17.1
Bhola	-18.1	-47.8	-41.2	-7.4	0.2	-13.9	-19.3	-18.2	-15.3	-2.7	-4.5	7.4	-15.5	-19.4
Jhalokathi	-1.2	-20.8	-64.9	-39.5	-3.9	-0.9	-5.6	-5.0	-6.4	-10.0	-9.1	-2.7	-10.6	-15.5
Patuakhali	-4.8	-28.2	-37.0	-16.5	-2.7	-5.3	-7.2	-6.9	1.9	-4.8	2.0	-1.7	-10.2	-12.0
Pirojpur	-3.8	-16.5	-43.9	-29.1	-4.6	-5.0	-10.8	-4.4	-5.5	-4.6	-9.4	-4.9	-4.3	-13.1
Bandarban	4.4	2.6	12.7	14.6	8.1	0.6	-2.1	0.3	3.0	0.1	-14.0	-25.6	-23.1	4.0
Brahmanbaria	-10.6	-19.9	-22.7	-12.5	-0.3	-1.8	5.8	1.7	3.8	-7.6	0.0	0.8	6.0	-7.8
Chandpur	-2.6	-10.1	-44.5	-29.8	-12.0	-1.5	-3.5	2.6	-5.0	-1.8	-1.8	6.4	0.3	-11.2
Chattogram	12.0	25.1	4.6	-17.3	-20.4	-8.7	-6.7	-1.4	-7.9	-1.3	-12.2	-12.8	-17.1	-0.3
Cumilla	6.0	-3.8	74.7	-14.3	-5.3	2.9	5.0	7.9	0.9	3.9	1.3	10.7	6.4	9.2
Cox’s Bazar	3.2	3.2	-5.5	-0.6	2.3	0.1	4.6	6.5	5.9	-1.6	0.4	-9.2	6.3	1.4
Feni	10.5	15.1	-15.8	-29.0	-9.7	0.4	4.6	5.0	6.9	2.2	10.3	7.7	9.0	0.3
Khagrachari	-4.7	-15.3	-6.7	9.9	7.9	-1.2	-7.1	-6.2	-6.9	-0.4	-12.3	5.3	-15.0	-3.6
Lakshmipur	-0.9	-9.5	-28.9	-14.8	-0.9	2.8	2.5	1.9	3.4	2.5	8.3	8.9	8.2	-4.8
Noakhali	4.5	-2.6	-22.8	-15.9	-5.4	2.2	8.9	9.9	3.9	5.0	13.7	7.8	11.0	-1.6
Rangamati	1.9	-4.8	1.4	5.8	7.9	1.2	-9.6	-7.3	-3.5	-2.0	-9.8	-13.2	-7.7	-0.5
Dhaka	29.9	55.8	53.7	34.6	6.9	1.5	1.8	7.5	1.8	7.2	-1.8	-0.3	-15.8	26.1
Faridpur	-3.0	-15.2	-25.7	-10.9	-1.7	-5.1	-2.2	-0.4	-1.1	-3.7	-0.3	7.5	-6.5	-7.4
Gazipur	27.8	56.8	75.4	96.7	40.8	121.9	24.6	18.8	18.1	9.0	11.2	7.8	4.9	53.9
Gopalganj	-9.5	-24.9	-44.2	-30.3	-8.8	-8.7	-6.0	-7.0	-13.4	-14.6	-10.8	1.0	-22.6	-17.6
Jamalpur	-13.1	-32.8	-28.3	-2.7	6.3	-3.2	-5.4	-8.8	-3.1	-1.3	-2.7	-10.1	-4.1	-10.1
Kishoreganj	-23.5	-28.4	-18.5	-1.7	-3.4	-6.1	-7.1	-7.3	-5.9	-6.6	-8.1	2.0	-0.2	-12.0
Madaripur	-8.1	-27.3	-51.0	-27.0	-2.3	-5.3	-2.2	-7.7	-3.6	-16.5	-4.3	-13.6	-16.5	-16.0
Manikganj	-1.6	-9.7	-18.4	-11.5	-3.1	1.5	3.3	1.2	4.2	-5.5	3.7	2.9	4.2	-3.9
Munshiganj	8.0	-11.1	-32.0	-34.2	-13.8	-6.2	0.4	0.1	3.1	-6.2	4.8	48.2	5.8	-7.7
Mymensingh	-19.0	-29.0	-13.4	4.7	1.7	-6.6	-4.2	-5.4	2.9	0.3	3.5	11.1	12.9	-7.2
Narayanganj	16.7	35.1	35.5	26.9	10.9	7.7	10.2	5.2	2.6	-5.7	9.0	-4.7	-6.4	18.5
Narsingdi	-4.9	-3.1	-5.0	0.6	0.7	-0.7	-0.3	-0.8	-1.3	-4.5	-6.3	-3.2	-11.1	-2.7
Netrokona	-22.0	-35.4	-24.0	-3.4	2.8	-9.3	0.2	-12.6	-0.5	-15.7	2.3	8.0	15.0	-11.3
Rajbari	-1.8	-10.1	-23.8	-9.4	3.2	0.1	0.2	3.0	0.9	6.5	-0.8	18.1	0.0	-4.1
Shariatpur	-5.7	-33.8	-53.1	-17.0	2.2	0.2	0.3	-2.5	-0.9	-2.8	1.5	4.4	21.6	-11.2
Sherpur	-16.6	-44.4	-25.8	2.5	-2.3	-14.4	-9.3	-16.9	-6.0	-9.8	-3.4	-8.8	5.9	-13.7
Tangail	-0.7	-12.0	-14.9	-9.1	-3.2	-2.6	4.7	-1.1	3.1	-1.5	7.1	-0.9	15.8	-3.3
Bagerhat	-8.6	-21.0	-36.3	-26.8	-18.0	-16.6	-9.1	-4.7	-13.9	-14.8	-11.1	-12.4	-9.6	-17.1
Chuadanga	9.2	2.0	-10.3	3.9	-1.0	-5.4	2.4	6.9	-1.9	3.1	-1.1	3.4	-2.8	0.8
Jashore	6.7	4.8	-6.6	-1.7	-4.0	-6.3	0.1	5.3	3.5	0.0	6.8	6.4	10.8	0.9
Jhenaidah	8.5	1.5	-11.1	-5.0	-3.0	-7.4	5.7	7.7	6.0	-3.2	9.1	1.8	14.7	0.6
Khulna	-5.2	-2.2	-12.2	-22.0	-19.8	-18.4	-14.3	-9.5	-19.1	-18.1	-16.5	-19.0	-5.9	-13.0
Kushtia	6.0	0.9	-10.1	-5.1	-2.3	-4.3	5.5	4.7	3.4	0.8	6.5	-0.6	0.3	-0.3
Magura	3.2	-8.5	-23.9	-21.0	17.8	2.6	20.2	33.6	25.1	44.9	-1.4	55.3	-64.9	3.2
Meherpur	8.0	8.7	-10.6	-4.3	-7.8	-3.0	5.7	11.1	0.1	7.6	1.6	-2.3	-2.1	0.5
Narail	-7.3	-18.6	-34.4	-24.7	-6.1	-8.6	-2.4	0.0	-5.9	-6.9	-6.6	-9.2	-9.5	-12.7
Satkhira	-3.3	-7.4	-11.3	-6.4	-12.1	-8.4	4.0	7.1	-0.5	-2.3	0.7	-5.6	6.1	-4.6
Bogura	-0.4	0.3	-2.0	6.4	0.9	0.4	-4.9	1.9	-2.5	8.5	-4.9	1.9	-9.1	0.1
Joypurhat	-4.9	-3.0	-3.8	2.6	1.8	-5.0	-2.1	-3.6	-1.4	-2.3	-2.5	-8.3	-9.2	-2.5
Naogaon	-8.8	-8.0	0.4	5.7	3.2	-6.3	-2.3	-2.6	1.7	-1.5	-1.3	-11.0	-3.7	-2.3
Natore	-0.8	-0.3	-4.3	0.1	-0.9	-2.0	1.6	0.6	4.2	3.2	6.7	1.9	11.8	0.2
C. Nawabganj	-0.9	-6.0	-9.2	-2.1	4.1	4.6	1.0	-0.1	3.5	10.0	-6.0	0.8	-9.7	-1.4
Pabna	2.0	-4.5	-8.9	-1.9	4.1	-0.6	4.5	-1.6	1.5	3.9	-1.8	-5.0	13.1	-0.3
Rajshahi	5.8	11.0	6.7	-0.2	-6.3	-3.9	4.4	2.0	1.8	-2.5	9.2	-4.4	7.2	2.9
Sirajganj	-7.1	-13.2	-6.5	-5.5	2.4	0.6	1.7	-4.2	1.5	3.9	3.1	-6.8	0.1	-3.8
Dinajpur	-6.3	-5.2	-5.4	2.1	4.6	-1.6	-1.2	-2.6	2.5	6.7	-1.8	-4.2	7.2	-1.4
Gaibandha	-12.6	-30.2	-16.5	4.5	7.5	-2.3	-6.6	-10.9	0.9	5.6	2.2	-7.3	-3.0	-7.1
Kurigram	-9.4	-26.9	-15.1	8.2	14.3	0.7	-1.1	-6.9	5.7	8.7	4.4	-1.5	18.1	-2.7
Lalmonirhat	-10.1	-23.9	-15.1	5.4	7.6	-3.1	-8.2	-5.8	-1.8	4.4	2.0	2.3	5.7	-5.5
Nilphamari	-11.0	-19.3	-8.1	10.5	9.2	3.6	-4.8	-2.1	-5.2	7.4	-1.6	0.8	-11.5	-3.4
Panchagarh	-7.4	-9.5	-8.5	8.6	13.6	5.6	0.1	0.4	1.0	9.8	-2.4	-0.4	-2.5	-0.2
Rangpur	-4.2	-10.9	-8.0	0.5	4.8	-3.1	-3.8	-3.0	0.2	11.6	1.8	0.1	4.8	-2.4
Thakurgaon	-8.6	-12.5	-9.5	2.8	10.3	1.2	-0.4	-5.1	-3.4	4.6	-2.2	-4.3	-1.9	-3.0
Habiganj	-6.6	-10.7	-9.3	-7.6	-4.7	-2.7	0.8	-4.5	-1.4	-13.1	-2.2	-18.5	0.6	-6.1
Maulvibazar	0.9	9.9	-6.0	-7.4	-3.3	2.2	3.0	1.1	-2.9	0.0	-0.6	-6.0	-8.5	-0.5
Sunamganj	-10.9	-12.8	-4.4	-5.3	-3.6	-5.6	3.8	-8.2	3.0	-15.4	6.8	-1.2	2.8	-5.4
Sylhet	10.2	25.5	13.8	0.6	-2.6	7.0	18.2	12.5	9.4	7.8	11.3	-1.5	4.9	10.6

The in-migration is the positive value (colored value), and out-migration is the negative value. The negative data mean those districts experience more out-migration than in-migration; the positive value means the other way. A higher value (positive or negative) indicates a higher migration rate.

## Results

### Characteristics of districts in Bangladesh

Bangladesh consists of 64 Districts and eight major regions (Division). Each district had distinct socioeconomic characteristics. As for 2011, from S1 Table (S2 Table for 1991 and 2001), Dhaka was the city having most urban population (77.3%) followed by Chattogram (41.4%), Khulna (33.5%), Narayanganj (33.5%), Rajshahi (32.9%), Gazipur (30.5%), Barishal (22.3%) Sylhet (21.9%), Mymensingh (15.6%) and Rangpur (15.3%).

The economically active population was the highest in Gazipur (61.2%), followed by Bandarban (60.2%), Dhaka (59.7%), and Narayanganj (56.1%). The literacy rate was also higher in divisional cities compared to others. Dhaka had the highest literacy rate of 70.5%, whereas Sunamganj had the lowest (34.9%) in 2011. Population Density was higher in divisional cities, which were also the industrial areas of Bangladesh. Dhaka, Gazipur, and Narayanganj are three districts having most of the readymade garments (RMGs), and other industries were most densely populated. Around 8299, 1884, and 4308 people lived per square kilometers in Dhaka, Gazipur, and Narayanganj. Never married male and female populations did not show much variation except for Sylhet, where both never-married males and females were the highest in Bangladesh (50.7% and 35.6%). Poverty was more elevated in coastal and northern districts than in others. Kurigram had the highest poverty headcount ratio (63.7%), followed by Barishal (54.8%), Shariatpur (52.6%), and Jamalpur (51.1%). Cities with more urban populations had lower poverty rate, such as Dhaka (15.7%), Gazipur (19.4%), and Chattogram (11.5%). The average household size was larger in districts with a less urban population, for example, Sunamganj (5.6), Maulvibazar (5.3), Brahmanbaria (5.3), and Feni (5.2), except for Sylhet (5.8).

For 2001, we also observed similar results as in 2011. Dhaka was the city having the most urban population percentage (91.6%), followed by Chattogram (51.1%), Narayanganj (56.2%), and Khulna (54%). The activity rate was also higher in the metropolitan cities, including Dhaka (48.2%), Gazipur (43.9%), Narayanganj (42.3%), and Netrokona (47.7%). The literacy rate was the highest in Jhalokathi (65.4%) and the lowest in Cox’s Bazar (30.8%). The population density was higher in the urban cities, like Dhaka (5814), Narayanganj (3106), Cumilla (1490), and Gazipur (1129). Never married male and female population percentage was higher in Sylhet (52.6% and 37.3%) and Chattogram (52.5% & 37.3%) and the lowest in eastern-northern part of Bangladesh, including Joypurhat (36.1% and 22%), Meherpur (37.1% and 22.3%). The average household size was the highest in Cox’s Bazar and Sylhet and the lowest in Bogura.

### Trends and patterns of internal migration

**Fig 2:**
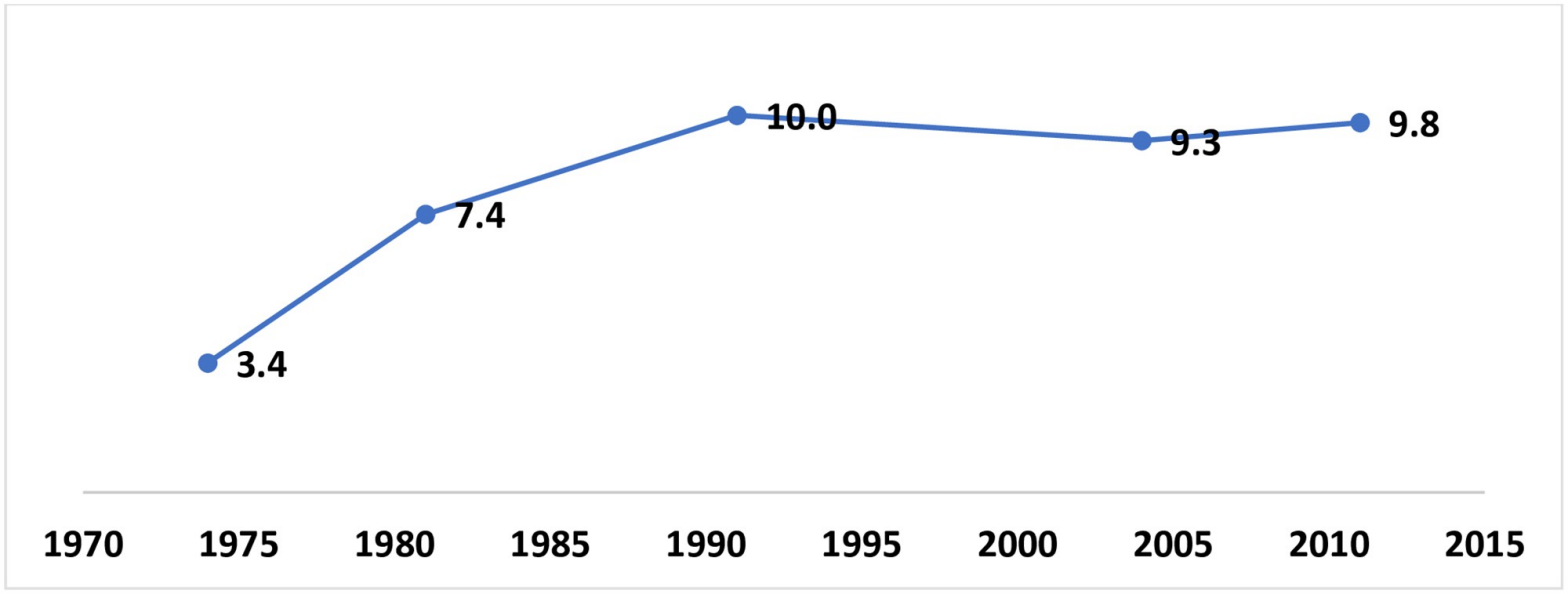
Trends (%) in lifetime internal migration in Bangladesh, 1974–2011.

Trends in lifetime internal migration in Bangladesh is present in Fig 2 (S1 and S3 Tables). The rate of lifetime internal migration showed an increasing trend from 1974 to 1991. Compared to previous, the rate slightly declined in 2004, and then it started increasing again in 2011.

Rural to rural and rural to urban migration was higher than urban to urban and urban to rural migration (S1 Table). Between 2001 and 2011, rural to rural migration was the highest in Gazipur, Dhaka, and Narayanganj (18.9%, 16.3%, and 14.1%). Between 1991–2001, we observed a similar kind of migration trend as of 2001–2011, where both within districts and outside districts, internal migration was higher for rural to rural. Dhaka, Gazipur, Narayanganj, Chapai Nawabganj, Chattogram, and Rangamati districts had a high internal migration rate.

The age-specific net migration rate between the 2001 and 2011 census year is revealed in Table 1. The in-migration (positive value) and out-migration (negative value) were the highest among 15–19, 20–24, 25–29, 30–34 and 35–39 age groups. Dhaka, Narayanganj, and Gazipur were the three districts receiving the most internal migrants. The rate was highest in the age group of 15–19 and 20–24 (55.8% and 53.7%) for Dhaka. Gazipur’s rate was highest in the 35–39 and 25–29 age groups (121.9% and 96.7%). Coastal districts situated at the estuary of southern Bangladesh were the primary source of internal migrants. Barguna, Jhalokathi, Bhola, and Barishal had the highest net out-migrants rate (negative). Age 20–24 was the foremost source of internal migration in Jhalokathi, Barishal, Bhola, and Pirojpur (-64.9%, -54.7%, -41.2%, and -43.9%).

**Fig 3:**
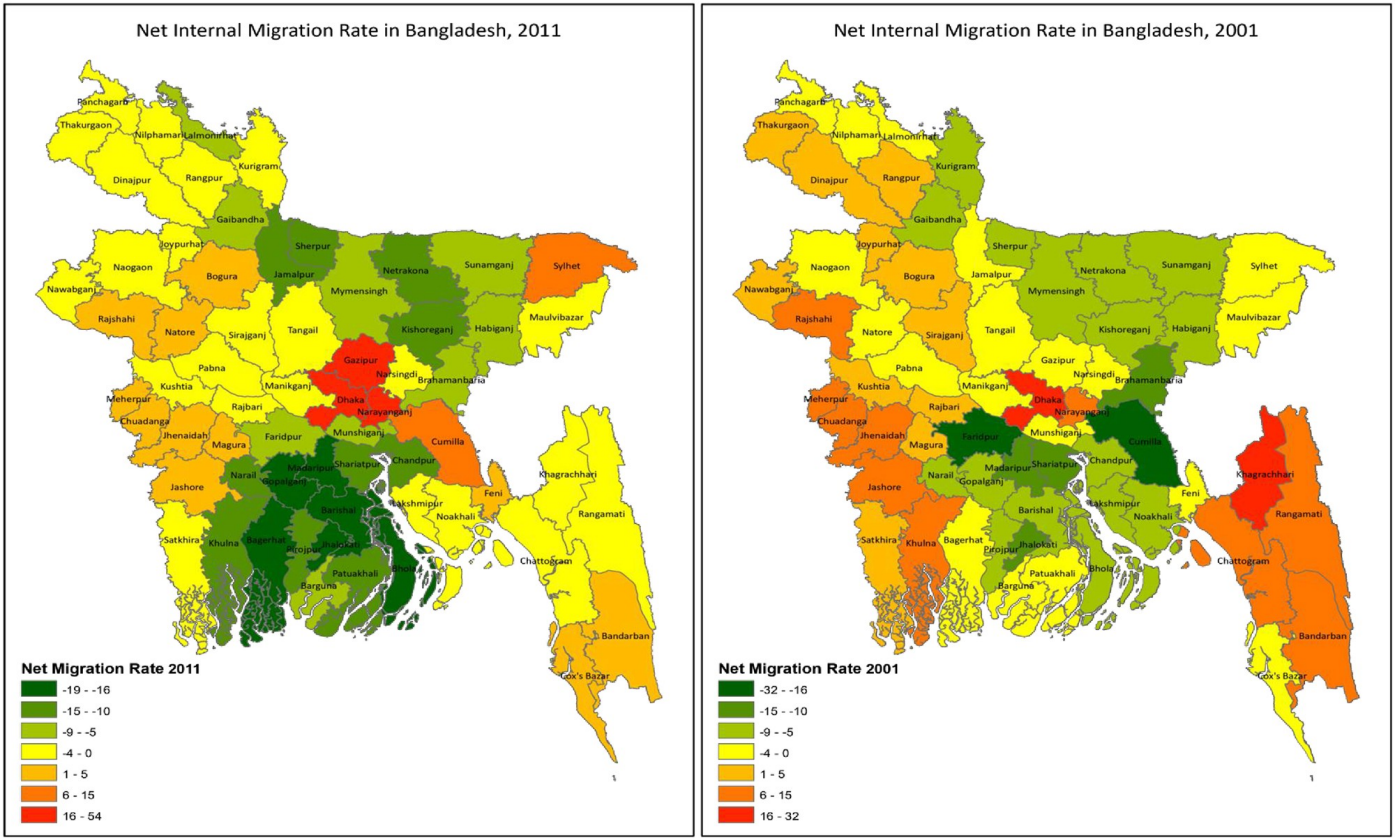
Net internal migration rate (%) in Bangladesh, 1991 to 2001 and 2001 to 2011. The maps were produced using ArcGIS (produced by authors). The maps presented the district-wise net migration rate based on indirect methods. The legends show negative migration to positive migration.

The distribution of internal migration is depicted in Fig 3 for 1991–2001 and 2001–2011. Dhaka, Narayanganj, and Gazipur were three districts with more than a 16% net migration rate. In contrast, Gazipur had less than 16% between 1991 and 2001. Other districts with a high migration rate for 1991–2001 were Chattogram, Khulna, Rajshahi, Rangamati, and Meherpur. The net out-migration rate was the highest in Bangladesh’s coastal and northern parts. Between 1991 and 2001, the highest net out-migration rate was observed in Faridpur (rate having between -32% and -25%), followed by Bhola, Barguna, Brahmanbaria, Jamalpur, and Sherpur (rate between -24% to -5%).

Between 2001 and 2011, net internal in-migration was higher in Dhaka, Naraynganj, and Gazipur. In contrast, Sylhet and Cumilla had a moderate in-migration rate between 6% and 15%. Net out-migration was high like 1991–2001 in the Coastal and Northern part of the country. Bhola, Netrokona, Kishoreganj, Jamalpur, and Sherpur had the highest net out-migration rate, higher than -19%. Districts of Khulna and Barishal division had internal out-migration rate between -18% to -10%.

The Divisional variation of net migration rate and relative changes between 2001 and 2011 is presented in Table 2. In 2001, Dhaka (3.5%), Khulna (3.2%), and Rajshahi Division (1.3%) had a positive net migration rate. However, positive net migration was observed only in Dhaka Division (7.4%) in 2011. The relative changes were positive (more in-migration) for Sylhet (125.4%), Dhaka (109.7%), and Chattogram Division (83.2%); the rest of the Divisions had negative relative changes (more out-migration).

**Table 2:** Total migrants, population, migration rate (%), and relative change (%) by division.

Division	2011			2001			Relative change (%)
	Migrants	Population	Rate (%)	Migrants	Population	Rate (%)	
**Barishal**	-923011	8325666	-11.1	-409561	8173718	-5.0	-121.3
**Chattogram**	-116236	28423019	-0.4	-590702	24290384	-2.4	83.2
**Dhaka**	2684897	36433505	7.4	1025678	29180051	3.5	109.7
**Mymensingh**	-714051	10990913	-6.5	-419560	9864665	-4.3	-52.8
**Khulna**	-527468	15687759	-3.4	465579	14705229	3.2	-206.2
**Rajshahi**	-104096	18484858	-0.6	210653	16354723	1.3	-143.7
**Rangpur**	-355984	15787758	-2.3	-105417	13847150	-0.8	-196.2
**Sylhet**	55950	9910219	0.6	-176671	7939343	-2.2	125.4

Note: Negative means more out-migration and positive means more in-migration.

**Fig 4:**
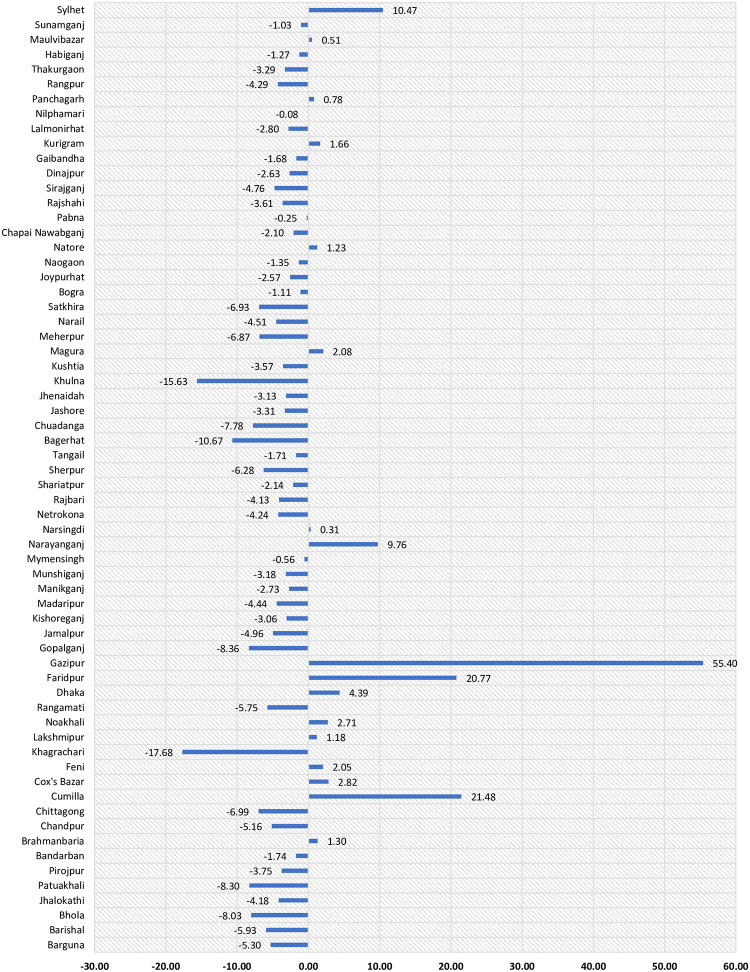
Relative change (%) of net internal migration between 2001 and 2011. Negative means more out-migration and positive means more in-migration.

Fig 4 shows the relative proportional change of net migration between 2001 (1991–2001) and 2011 (2001–2011). The relative changes were positive (more in-migration) for Gazipur, Comilla, Faridpur, Sylhet, Narayanganj, Dhaka, Cox’s Bazar, Noakhali, Magura, Feni, Kurigram, Brahmanbaria, Natore, Lakshmipur, Panchagarh, Maulvibazar, and Narsingdi districts. At the same time, the rest of the districts had negative relative changes in net migration (more out-migration) between 2001 and 2011.

### Determinants of internal migration

Table 3 shows the correlation between socioeconomic factors and net internal migration (indirect) based on the 2011 census data. The correlation coefficient was the strongest with the activity rate (r = 0.616, p<0.001) followed by population density per square km (r = 0.508, p<0.001), urbanization rate (r = 0.503, p<0.001), city Corporation (r = 0.485, p<0.001), literacy rate (r = 0.191, p = 0.130), percentage of never-married male (r = 0.102, p = 0.423), percentage of never-married female (r = 0.084, p = 0.509). In contrast, the net migration rate was negatively correlated with the poverty rate (r = -0.379, p = 0.002) and average household size (r = -0.015, p<0.905).

**Table 3:** Correlation between socioeconomic factors and internal migration in Bangladesh, 2011.

	CC	UR	AR	LR	PD	NMM	NMF	PR	AH	NMR2011
CC	1									
UR	0.608***	1								
AR	0.281*	0.563***	1							
LR	0.424***	0.403***	-0.056	1						
PD	0.450***	0.717***	0.395***	0.374**	1					
NMM	0.289*	0.268*	-0.063	0.056	0.194	1				
NMF	0.215	0.275*	0.123	-0.002	0.126	0.924***	1			
PR	-0.060	-0.239	-0.204	-0.147	-0.185	-0.060	-0.067	1		
AH	0.064	0.020	-0.110	-0.149	-0.043	0.857***	0.824***	-0.123	1	
NMR2011	0.485***	0.503***	0.616***	0.191	0.508***	0.102	0.084	-0.379**	-0.015	1

NMR2011: Net internal migration rate 2011, UR: Urbanization rate, AR: Activity rate; LR: Literacy rate; PD: Population density per square mile; NMM: Percentage of never-married male; NMF: Percentage of never-married female; HS: Average household size; CC: City corporation (dummy); PR: Poverty rate.

***P≤0.001,

**P≤0.01,

*P≤0.05.

The socioeconomic factors were then entered into a stepwise multiple linear regression model to determine the predictors of internal migration. Due to multicollinearity, the urbanization rate and the proportion of never-married (VIF >10) were excluded from the regression model. We excluded population and average household as insignificant at the bivariate analysis. Table 4 shows the stepwise multiple regression analyses of the net migration rate of 2011 and the lifetime internal migration rate of 1991, 2001, and 2011. For net internal migration 2011, activity rate was the strongest predictor (β = 0.419, P<0.001). The city corporation was the central hub of migration (β = 0.275, P<0.01). On the other hand, the district with a higher poverty rate had lower internal migration (β = -0.246, P<0.01). The coefficient of lifetime internal migration rate was almost identical to the net migration rate of 2011.

**Table 4:** Socioeconomic factors affecting internal migration in Bangladesh.

SEC	NMR, 2011		LTIM, 2011		LTIM, 2001		LTIM, 1991	LTIM, All
	β (95% CI)		β (95% CI)		β (95% CI)		β (95% CI)	β (95% CI)
	1	2	1	2	1	2	1	1
AR	0.419 [0.51, 1.44]***	0.467 [0.66, 1.51]***	0.596 [0.89, 1.20]***	0.619 [0.93, 1.24]***	0.225 [0.02, 0.79]*	0.186 [-0.04, 0.71]*	0.235 [0.12, 0.35]***	0.408 [0.45, 0.75]***
LR	-0.003 [-0.30, 0.29]		0.393 [0.32, 0.51]***	0.418 [0.35, 0.54]***	0.139 [-0.11, 0.40]		0.161 [0.01, 0.12]*	0.341 [0.13, 0.33]***
PD	0.174 [0.01, 0.44]		0.320 [0.001, 0.003]***	0.335 [0.001, 0.003]***	0.430 [0.002, 0.007]***	0.526 [0.004, 0.008]***	0.432 [0.002, 0.004]***	0.386 [0.002, 0.004]***
CC	0.275 [1.98, 14.3]**	0.338 [4.64, 15.3]***	0.046 [-1.04, 3.1]		0.140 [-2.6, 10.9]		0.528 [5.4, 8.4]***	0.139 [0.91, 5.9]**
PR	-0.246 [-0.38, -0.06]**	-0.264 [-0.39, -0.08]***	-0.069 [-0.10, 0.01]					
Year: 1991^RC^								
2001								0.281 [2.4, 6.4]***
2011								-0.130 [-4.7, 0.43]
**Model Summary**								
R^2^	0.572	0.551	0.915	0.915	0.381	0.336	0.856	0.642
Adjusted R^2^	0.535	0.529	0.908	0.908	0.339	0.314	0.846	0.631

SEC: Socioeconomic characteristics; AR: Activity rate; LR: Literacy rate; PD: Population density per square mile; CC: City corporation (dummy); PR: Poverty rate. RC: Reference category; NMR: Net internal migration rate; LTIM: Lifetime internal migration rate. 1: Full model; 2: Reduced model.

RC: Reference category. β = Standardized beta coefficients; 95% CI = 95% confidence intervals in brackets (unstandardized beta coefficients).

***P≤0.001,

**P≤0.01,

*P≤0.05.

We observed a similar result for the lifetime internal migration rate in 2001 and 1991. However, the strongest predictor of migration in 2001 and 1991 was population density (β = 0.526, P<0.001) and City Corporation (β = 0.528, P<0.001). An overall model for lifetime internal migration showed that activity rate (β = 0.408, P<0.001), population density (β = 0.386, P<0.001), literacy rate (β = 0.341, P<0.001), and city corporation (β = 0.139, P<0.01) were the significant factors of internal migration rate.

### Causes of internal migration

Table 5 shows the overall causes of the lifetime internal migration in Bangladesh. More than 38% of migration resulted from marriages, ranging between 25.6% in Dhaka and 63.6% in the Khulna division. Around 29% (16.2% for employment/business and 12.8% for looking for a job) of migration resulted from economic reasons. People tended to migrate from minor to more diversified economic opportunities areas. Education was responsible for 4.9% of migration. Moreover, natural disasters played a vital role in migration in the Rangpur division (10.9%).

**Table 5:** Causes of internal migration: Push-pull factors, 2011.

Causes of migration	Barishal	Chattogram	Dhaka	Khulna	Rajshahi	Rangpur	Sylhet	Total
**Pull Factors**								
Marriage	50.5	30.9	25.6	63.6	43.2	55.5	32.3	38.7
Education	8.4	4.2	5.3	3.7	6.6	1.8	4.5	4.9
Employment/business	16.1	17.9	21.2	7.6	13.4	7.6	19.0	16.2
Looking for job	7.3	16.5	15.2	5.9	5.0	7.1	27.9	12.8
**Push Factors**								
Natural calamity	0.6	4.3	0.9	1.4	3.0	10.9	0.1	2.1
Family problems	1.1	2.1	1.1	1.1	1.3	1.7	2.6	1.6
**Others**	16.0	24.1	30.7	16.7	27.5	15.5	13.7	23.7

## Discussion

This study aimed to understand the trends, patterns, and determinants of internal migration in Bangladesh based on the census data from 1991 to 2011. By analyzing the internal migration rate for 2001 and 2011, the study found that the number of net migrants was higher in the more urbanized city like Dhaka, Narayanganj, Gazipur, and Chattogram. The net migration rate was also higher in the divisional town than in other districts. Cities with more industries and more economic opportunities had a higher net in-migration rate; on the other hand, cities with higher poverty headcount ratio, coastal areas had a higher negative rate of internal migration. These findings are similar to a study conducted in Indonesia [46]. Analysis of census data from 1930 to 2011 in Indonesia showed that urban primacy was a striking force for migration streams. The report of UNDP on internal migration also showed a similar interpretation as our finding, where they showed that most of the migrants migrated to Dhaka, Gazipur, and Chattogram [8].

In terms of age, the 20–39 age group had the highest internal migration rate (both in and out-migration). The IMAGE project also found that among Asian countries, migration intensity tends to peak at the 20s with a variation for Japan, which has a developed socioeconomic structure and comparatively aging population [29]. The characteristics of this age group are quite distinct as they (both male and female) were either in education or in employment. Migration and education had a complex relation. Migrants are inclined to be more educated than the general population; then again, many internal migrations occur for education [47]. Students from rural areas or other cities come to Dhaka, Gazipur, Chattogram, and divisional metropolises, where many public and private universities, schools, and colleges have been situated to pursue higher education. Apart from education, many people of this age group also migrate to look for jobs, join new jobs, or transfer current jobs [21, 48]. Marriage, a significant source of internal migration for females [10], could also explain the higher migration rate in this age group.

In Bangladesh, the rural to rural and rural to urban migration rate was higher due to the inequality in development in the rural and urban sectors. In this regard, urban bias plays an essential role in developing countries. Urban bias interprets that most administrations in developing countries, for example, Bangladesh, favor the urban sectors in their development policies, making a large gap between the rural and urban economies [11]. For instance, internal migration can be called ‘urban migration’ in many developing and less developed countries [48]. Zelinsky’s hypothesis also states that Bangladesh, being at the third stage of demographic transition, experiences higher rural to urban migration [37]. Evidence from Italy shows that their internal mobility rate is falling every year. Every state and city is well developed; thus, people do not need to migrate searching better life [49]. However, there has been a substantial difference between urban and rural sectors regarding employment, lifestyle, and income in Bangladesh. As a result, rural to urban and rural migration is higher.

We observed that economic reasons are the major causes of migration. People migrated for jobs or employment. In line with this, Todaro and Smith also found the movement of people from rural villages and peripheries to urban centers in search of jobs [11]. The IMAGE project also identified that economic reasons were the primary cause behind internal migration in Thailand, India, and Nepal [29]. Population growth in the rural area and less availability of working opportunities lead people to move to a place where more jobs are available; from a developing country perspective, urban and divisional cities hold such characteristics [48].

Moreover, many of the population come to city corporation areas in Bangladesh. The notion of first-city, a form of urban bias, is responsible for this. The country’s first or largest city usually gets a substantial share of public and private investment and incentives than the second-largest city or other smaller cities. As a result, the first city receives a disproportionately and unproductively large population. Dhaka, the capital and first city, is central to any investment in Bangladesh, contributing 40% to the country’s total GDP, followed by Chattogram (the Second highest contributor in the GDP). In this way, we found that Dhaka and Chattogram are the two central areas of receiving migrants.

We found that the activity rate was the significant predictor of both the net and lifetime internal migration. This finding is also supported by the studies from Indonesia and India- where employment and business were the striking force behind internal migration [46, 50]. Based on lifetime internal migration data, BBS also showed that most internal migration occurs due to employment and business [10]. The literacy rate is another crucial determinant of internal migration. As literate people tend to go for better job opportunities and a better lifestyle, the rate was higher among districts with a higher literacy rate [21]. The paper also showed a strong correlation between marriage and internal migration. The correlation was even higher among females, as they usually change their residence after marriage. These two findings are similar to the Population Monograph of Bangladesh [10], and forming family/marriage was also identified as predictors of internal migration in India and Nepal by the IMAGE project [29].

There is a negative association between the poverty rate and migration. Theoretically, poverty creates the necessity for migration [30, 48]. People living in poor cities try to move to a place where the lifestyle is more developed, and the employment opportunity is even higher than others. Todaro’s model elucidates rural-urban migration as an economically rational process notwithstanding high urban unemployment. Migrants habitually calculate expected gains from the migration decision, calculated by the difference between rural and urban real wages and the probability of getting a job in the urban sector [11, 51]. The context of poverty is clearly stated in the new economics of migration since the family decides migration to minimize the risk due to poverty [32]. In Bangladesh, the rural employment sector is mainly agriculture-based, whereas the urban job sector is multidimensional. Therefore, working in various professions and getting more wages by working as much as in the rural economy shapes people’s decision from the northern and coastal areas of Bangladesh to migrate to the urban industrial economy despite the high urban unemployment rate [51]. Coastal districts (districts from Barishal and Khulna division) are situated in southern Bangladesh [52]; those are highly vulnerable to floods and cyclones, which act as push factors in migrating.

On the other hand, the Harris-Todaro model predicts that expected incomes are generally equated across rural and urban sectors when considering informal-sector activities and outright unemployment. The rural sector can implement all its labor in agriculture (only job sector in rural) or send the surplus to the urban specialized job sector where the migration decision depends on the expected real income in urban sector times unemployment ratio [11, 31]. In this regard, migration decision is economically rational. In the case of high unemployment, labor goes into informal sectors and the labor migrated from rural have ties with their place of origin, so that the money is sent to the rural sector and it would develop [31]. In Bangladesh, the rural surplus labor decides to migrate because wages in urban areas in Bangladesh are much higher and job opportunities are high. Therefore, rural people migrate to urban industrial cities.

We also found that 3% of migration took place due to natural calamities (flood, drought, river erosion, water salinity, and many more), which was around 11% in the Rangpur division, followed by Chattogram (4.3%) and Rajshahi (3.0%). Rajshahi and Rangpur is the northern region of Bangladesh. The population of the northern region is severely affected by flood and drought in the rainy and winter season, respectively. As a result, migration is a tradeoff for them to live [20]. In Chattogram division, the population mostly suffers from cyclones and landslides in the hill track. Therefore, migration is only the option left to avoid the grieve consequences of cyclones and soil erosion of hills.

## Conclusion

The study of internal migration is of particular need to understand a country’s current and future population distribution. Due to the dearth of rigorous studies regarding internal migration in Bangladesh, we attempted to understand internal migration trends, patterns, and determinants using Population and Housing Census data from 1991 to 2011. We observed that rural to urban migration is still ahead of urban to rural migration, and migration occurs mainly for economic reasons. The destination of migration is a few developed metropolitan cities (e.g., Dhaka, Gazipur, Narayanganj, and all the Divisional cities). In contrast, the areas of origin of migration are poor, less developed, and coastal districts. This finding has important policy implications. If the current migration trends continue, few developed cities will have an excessive population, which will increase population density and pollution, thereby decreasing living standards. There are disproportionate differences between metropolitan and other cities in terms of opportunities, which is one reason for having many migrants in those cities. Therefore, along with comprehensive urban planning, decentralization of government and private institutions must be ensured. To reduce urban migration, as stated in various policies and programs of Bangladesh, we also suggest that new employment opportunities should be created at the area of origin as economic activity is one of the key reasons for migration. This study also demonstrates that the working-age population has a higher migration rate; so, education, training, and work opportunities for the migrants should be guaranteed in the area of origin.

This study showed dynamics of internal migration using both indirect (using UN Manual) and direct methods to estimate internal migration data from population and housing census. As a result, this study becomes more credible in understanding the depths and patterns of internal migration. Since we appraised the migration using the balancing equation of population data, there would be both estimation error and content error. Moreover, the lifetime internal migration is calculated based on the ‘place of birth’ data, and indirect estimation is also based on place of birth and current place of residence; thus, they do not take account of timing and number of migrations that occurred during the inter-censual period. In this circumstance, the data from the vital registration system would be more credible and accurate as it collects data every year. However, with the unavailability of raw data, the vital registration system is not robust in Bangladesh, which leaves us no choice but to use these statistics. Some crucial determinants of migration, for example, specific natural calamity, the distance between the destination and current place of residence, could not be analyzed due to the unavailability of data. This study utilized the data from the macro-level (district as the respondents); however, the micro-level analysis would be more precise and accurate. Despite all those limitations, the study’s findings would help understand Bangladesh’s population dynamics and the relationship between development and migration.

## Supporting information

S1 Table Lifetime internal migration and socioeconomic characteristics by districts of Bangladesh, 2011. (DOCX)

S2 Table Socioeconomic characteristics of the districts of Bangladesh, 1991 and 2001. (DOCX)

S3 Table Lifetime internal migration by districts of Bangladesh, 1991 and 2001. (DOCX)
