# Supplementary material for: Dynamics of internal migration in Bangladesh: Trends, patterns, determinants, and causes
Source: PLoS One. 2022 Feb 14;17(2):e0263878. doi: 10.1371/journal.pone.0263878 (PMC8843202; doi:10.1371/journal.pone.0263878)
Supplement: S1 Table — (DOCX) [file pone.0263878.s001.docx]

**S1 Table.** Lifetime internal migration and socioeconomic characteristics by districts of Bangladesh, 2011

| **District** | **Life Time Migrants (%)** | | | | | **Socioeconomic characteristics (%)** | | | | | | | |
| --- | --- | --- | --- | --- | --- | --- | --- | --- | --- | --- | --- | --- | --- |
|  | **RtR** | **RtU** | **UtR** | **UtU** | **Total** | **UR** | **AR** | **LR** | **PD** | **NMM** | **NMF** | **PR** | **HS** |
| Barguna | 3.2 | 0.8 | 0.4 | 0.2 | 4.6 | 11.5 | 42.3 | 57.6 | 488 | 32.7 | 20.1 | 19.0 | 4.1 |
| Barishal | 1.1 | 1.7 | 0.3 | 0.6 | 3.7 | 22.3 | 41.9 | 61.2 | 835 | 40.2 | 26.9 | 54.8 | 4.5 |
| Bhola | 0.5 | 0.2 | 0.2 | 0.1 | 1.0 | 13.7 | 45.2 | 43.2 | 522 | 38.6 | 26.3 | 33.2 | 4.8 |
| Jhalokathi | 3.8 | 0.6 | 1.0 | 1.1 | 6.5 | 16.4 | 38.9 | 66.7 | 966 | 39.2 | 25.4 | 40.5 | 4.3 |
| Patuakhali | 1.7 | 0.4 | 0.1 | 0.2 | 2.4 | 13.1 | 43.2 | 54.1 | 477 | 35.2 | 22.6 | 25.8 | 4.4 |
| Pirojpur | 3.2 | 1.0 | 0.2 | 0.2 | 4.6 | 16.4 | 41.6 | 64.8 | 871 | 37.7 | 23.7 | 44.1 | 4.3 |
| Bandarban | 7.2 | 2.6 | 1.0 | 0.7 | 11.5 | 25.9 | 60.2 | 35.8 | 87 | 38.3 | 29.3 | 40.1 | 4.9 |
| Brahmanbaria | 1.6 | 0.3 | 0.3 | 0.3 | 2.5 | 15.8 | 39.7 | 45.3 | 1510 | 42.9 | 27.5 | 30.0 | 5.3 |
| Chandpur | 1.7 | 0.6 | 0.4 | 0.1 | 2.8 | 18.1 | 37.8 | 56.8 | 1468 | 43.2 | 27.9 | 51.0 | 4.8 |
| Chattogram | 1.4 | 8.4 | 0.1 | 1.7 | 11.6 | 41.4 | 46.8 | 58.9 | 1442 | 47.3 | 32.4 | 11.5 | 5.0 |
| Cumilla | 1.5 | 0.4 | 0.2 | 0.3 | 2.4 | 15.6 | 38.9 | 53.3 | 1712 | 43.1 | 27.6 | 37.9 | 5.1 |
| Cox's Bazar | 0.4 | 0.2 | 0.1 | 0.3 | 1.0 | 21.8 | 48.4 | 39.3 | 919 | 47.6 | 34.3 | 32.7 | 5.5 |
| Feni | 1.3 | 2.7 | 0.3 | 0.3 | 4.6 | 20.4 | 37.6 | 59.6 | 1451 | 47.1 | 28.8 | 25.9 | 5.2 |
| Khagrachari | 6.1 | 4.8 | 0.3 | 1.3 | 12.5 | 35.1 | 51.1 | 46.1 | 223 | 37.9 | 28.1 | 25.5 | 4.6 |
| Lakshmipur | 1.0 | 0.3 | 0.2 | 0.2 | 1.7 | 15.2 | 38.9 | 49.4 | 1200 | 43.1 | 27.2 | 31.2 | 4.7 |
| Noakhali | 1.3 | 0.2 | 0.2 | 0.1 | 1.8 | 15.9 | 37.9 | 51.3 | 843 | 45.6 | 29.6 | 9.6 | 5.2 |
| Rangamati | 6.7 | 2.6 | 0.5 | 0.7 | 10.5 | 26.8 | 53.6 | 49.7 | 97 | 38.8 | 28.9 | 20.3 | 4.6 |
| Dhaka | 16.3 | 29.5 | 0.7 | 4.3 | 50.8 | 77.3 | 59.7 | 70.5 | 8229 | 42.4 | 28.5 | 15.7 | 4.3 |
| Faridpur | 3.5 | 0.6 | 0.3 | 0.7 | 5.1 | 14.2 | 43.7 | 48.9 | 932 | 38.7 | 24.8 | 36.3 | 4.6 |
| Gazipur | 18.9 | 17.5 | 1.8 | 2.8 | 41.0 | 30.5 | 61.2 | 62.6 | 1884 | 36.7 | 23.3 | 19.4 | 4.1 |
| Gopalganj | 3.9 | 0.5 | 0.2 | 0.3 | 4.9 | 10.9 | 42.4 | 58.1 | 796 | 33.3 | 20.6 | 42.7 | 4.7 |
| Jamalpur | 2.0 | 0.4 | 0.1 | 0.2 | 2.7 | 16.9 | 44.5 | 38.4 | 1084 | 41.2 | 26.2 | 51.1 | 4.1 |
| Kishoreganj | 0.8 | 1.3 | 0.1 | 0.4 | 2.6 | 16.8 | 44.6 | 40.8 | 1083 | 39.7 | 25.5 | 30.3 | 4.6 |
| Madaripur | 1.9 | 0.4 | 0.6 | 0.3 | 3.2 | 13.5 | 44.5 | 47.9 | 1036 | 40.7 | 26.9 | 34.9 | 4.6 |
| Manikganj | 2.8 | 0.1 | 0.3 | 0.4 | 3.6 | 9.2 | 42.4 | 49.2 | 1007 | 34.2 | 20.1 | 18.5 | 4.3 |
| Munshiganj | 4.5 | 1.9 | 2.0 | 0.4 | 8.8 | 12.8 | 45.1 | 56.1 | 1439 | 36.5 | 24.2 | 28.7 | 4.6 |
| Mymensingh | 2.2 | 1.1 | 0.3 | 0.4 | 4.0 | 15.6 | 45.5 | 43.5 | 1163 | 40.1 | 26.4 | 50.5 | 4.4 |
| Narayanganj | 14.1 | 7.4 | 1.0 | 1.6 | 24.1 | 33.5 | 56.1 | 57.1 | 4308 | 40.4 | 25.7 | 26.1 | 4.4 |
| Narsingdi | 3.8 | 3.2 | 0.4 | 0.6 | 8.0 | 20.1 | 45.3 | 49.6 | 1934 | 39.4 | 25.1 | 23.7 | 4.7 |
| Netrokona | 1.7 | 0.4 | 0.1 | 0.2 | 2.4 | 11.1 | 46.5 | 39.4 | 798 | 36.0 | 22.8 | 35.3 | 4.7 |
| Rajbari | 6.3 | 1.1 | 0.8 | 0.6 | 8.8 | 12.9 | 43.8 | 52.3 | 961 | 40.8 | 25.9 | 41.9 | 4.4 |
| Shariatpur | 1.7 | 0.1 | 0.6 | 0.1 | 2.5 | 11.3 | 43.7 | 47.3 | 984 | 41.5 | 28.7 | 52.6 | 4.7 |
| Sherpur | 1.3 | 0.2 | 0.1 | 0.1 | 1.7 | 13.8 | 45.5 | 37.9 | 995 | 33.5 | 21.4 | 48.4 | 4.0 |
| Tangail | 1.1 | 0.2 | 0.3 | 0.1 | 1.7 | 15.1 | 42.7 | 46.8 | 1056 | 33.9 | 19.5 | 29.7 | 4.1 |
| Bagerhat | 3.5 | 0.9 | 0.6 | 0.8 | 5.8 | 13.2 | 43.9 | 58.9 | 373 | 35.4 | 22.7 | 42.8 | 4.2 |
| Chuadanga | 4.2 | 2.6 | 0.3 | 0.6 | 7.7 | 27.1 | 46.1 | 45.9 | 962 | 32.4 | 19.5 | 27.7 | 4.1 |
| Jashore | 5.4 | 2.9 | 0.4 | 0.7 | 9.4 | 18.6 | 44.2 | 56.5 | 1060 | 35.2 | 21.6 | 39.0 | 4.2 |
| Jhenaidah | 5.1 | 1.6 | 0.6 | 1.0 | 8.3 | 15.8 | 44.6 | 48.4 | 902 | 34.0 | 20.8 | 24.7 | 4.2 |
| Khulna | 4.6 | 7.7 | 0.2 | 1.3 | 13.8 | 33.5 | 44.9 | 60.1 | 528 | 37.5 | 23.2 | 38.8 | 4.2 |
| Kushtia | 4.7 | 1.1 | 0.3 | 0.4 | 6.5 | 12.1 | 44.6 | 46.3 | 1210 | 34.0 | 20.4 | 3.6 | 4.1 |
| Magura | 6.5 | 1.2 | 0.4 | 0.4 | 8.5 | 13.1 | 42.9 | 50.6 | 884 | 35.9 | 22.7 | 45.4 | 4.5 |
| Meherpur | 3.0 | 0.6 | 0.3 | 0.4 | 4.3 | 12.7 | 44.4 | 46.2 | 884 | 31.1 | 17.7 | 15.2 | 3.9 |
| Narail | 6.2 | 1.6 | 0.5 | 0.2 | 8.5 | 15.5 | 42.3 | 61.3 | 746 | 37.1 | 22.5 | 20.0 | 4.4 |
| Satkhira | 1.5 | 0.4 | 0.1 | 0.1 | 2.1 | 9.9 | 43.4 | 52.1 | 520 | 34.8 | 21.3 | 46.3 | 4.2 |
| Bogura | 3.5 | 1.0 | 0.5 | 0.8 | 5.8 | 19.7 | 44.9 | 49.4 | 1173 | 33.1 | 22.1 | 16.6 | 3.9 |
| Joypurhat | 5.8 | 2.2 | 0.4 | 0.5 | 8.9 | 15.7 | 44.8 | 57.5 | 903 | 31.3 | 23.3 | 25.3 | 3.8 |
| Naogaon | 2.8 | 0.4 | 0.1 | 0.1 | 3.4 | 10.6 | 45.5 | 48.2 | 757 | 31.8 | 19.6 | 26.7 | 4.0 |
| Natore | 4.2 | 1.4 | 0.7 | 0.4 | 6.7 | 13.3 | 44.6 | 49.6 | 898 | 32.9 | 18.2 | 16.9 | 4.0 |
| Chapai Nawabganj | 1.4 | 0.6 | 0.1 | 0.3 | 2.4 | 19.4 | 44.2 | 42.9 | 968 | 35.4 | 18.2 | 35.1 | 4.6 |
| Pabna | 1.9 | 1.0 | 0.2 | 0.5 | 3.6 | 15.3 | 45.3 | 46.7 | 1062 | 35.0 | 22.3 | 31.5 | 4.3 |
| Rajshahi | 1.9 | 3.9 | 0.2 | 1.2 | 7.2 | 32.9 | 43.2 | 52.9 | 1070 | 36.4 | 19.2 | 31.4 | 4.1 |
| Sirajganj | 1.0 | 0.3 | 0.1 | 0.2 | 1.6 | 14.1 | 45.6 | 42.1 | 1290 | 34.8 | 22.0 | 38.7 | 4.3 |
| Dinajpur | 3.8 | 1.1 | 0.2 | 0.3 | 5.4 | 15.2 | 46.1 | 52.4 | 868 | 35.1 | 21.7 | 37.9 | 4.2 |
| Gaibandha | 1.9 | 0.8 | 0.1 | 0.1 | 2.9 | 8.8 | 44.2 | 42.8 | 1125 | 32.7 | 19.7 | 48.0 | 3.9 |
| Kurigram | 0.9 | 0.2 | 0.3 | 0.4 | 1.8 | 15.8 | 43.5 | 42.5 | 922 | 34.0 | 19.8 | 63.7 | 4.1 |
| Lalmonirhat | 3.0 | 0.5 | 0.3 | 0.2 | 4.0 | 10.2 | 46.3 | 46.1 | 1007 | 35.4 | 22.6 | 34.5 | 4.3 |
| Nilphamari | 2.3 | 1.2 | 0.2 | 0.7 | 4.4 | 15.8 | 46.2 | 44.4 | 1186 | 35.8 | 23.7 | 34.8 | 4.4 |
| Panchagarh | 5.1 | 0.5 | 0.4 | 0.3 | 6.3 | 9.6 | 46.6 | 51.7 | 703 | 36.2 | 23.9 | 26.7 | 4.3 |
| Rangpur | 2.5 | 1.0 | 0.1 | 0.4 | 4.0 | 15.3 | 45.2 | 48.5 | 1200 | 35.3 | 22.1 | 46.2 | 4.0 |
| Thakurgaon | 3.5 | 0.7 | 0.3 | 0.5 | 5.0 | 11.6 | 45.2 | 48.7 | 780 | 35.9 | 23.7 | 27.0 | 4.3 |
| Habiganj | 3.3 | 0.2 | 0.2 | 0.1 | 3.8 | 11.7 | 45.7 | 40.5 | 792 | 42.9 | 29.2 | 25.3 | 5.3 |
| Maulvibazar | 3.0 | 1.1 | 0.4 | 0.2 | 4.7 | 10.8 | 45.4 | 51.1 | 686 | 45.9 | 31.9 | 25.7 | 5.3 |
| Sunamganj | 2.1 | 0.3 | 0.1 | 0.2 | 2.7 | 10.3 | 46.8 | 34.9 | 659 | 45.0 | 30.9 | 26.0 | 5.6 |
| Sylhet | 2.2 | 4.0 | 0.1 | 0.8 | 7.1 | 21.9 | 42.9 | 51.2 | 995 | 50.7 | 35.6 | 24.1 | 5.8 |

Note: RtR: Rural to rural; RtU: Rural to urban; UtR: Urban to rural; UtU: Urban to urban; UR: Urbanization rate, AR: Activity rate; LR: Literacy rate; PD: Population density per square mile; NMM: Percentage of never-married male; NMF: percentage of never-married female; PR: Poverty headcount ratio; HS: Average household size.
