# Supplementary material for: Dynamics of internal migration in Bangladesh: Trends, patterns, determinants, and causes
Source: PLoS One. 2022 Feb 14;17(2):e0263878. doi: 10.1371/journal.pone.0263878 (PMC8843202; doi:10.1371/journal.pone.0263878)
Supplement: S2 Table — (DOCX) [file pone.0263878.s002.docx]

**S2 Table.** Socioeconomic characteristics of the districts of Bangladesh, 1991 and 2001

| **District** | **Socioeconomic characteristics, 1991** | | | | | | | **Socioeconomic characteristics, 2001** | | | | | | |
| --- | --- | --- | --- | --- | --- | --- | --- | --- | --- | --- | --- | --- | --- | --- |
|  | **UR** | **AR** | **LR** | **PD** | **NMM** | **NMF** | **HS** | **UR** | **AR** | **LR** | **PD** | **NMM** | **NMF** | **HS** |
| Barguna | 8.7 | 40.0 | 40.1 | 424 | 40.5 | 25.3 | 5.2 | 10.3 | 38.3 | 55.3 | 463 | 39.7 | 25.7 | 4.7 |
| Barishal | 15.0 | 39.6 | 43.0 | 791 | 43.7 | 27.3 | 5.3 | 16.7 | 36.5 | 57.0 | 846 | 45.3 | 31.1 | 5.0 |
| Bhola | 12.8 | 43.7 | 24.0 | 434 | 39.6 | 25.7 | 5.5 | 13.8 | 38.7 | 36.9 | 500 | 41.2 | 29.2 | 5.2 |
| Jhalokathi | 12.8 | 37.4 | 51.1 | 879 | 44.8 | 27.6 | 5.2 | 15.0 | 34.8 | 65.4 | 927 | 44.0 | 28.8 | 4.8 |
| Patuakhali | 10.4 | 40.4 | 36.4 | 398 | 40.9 | 26.0 | 5.6 | 12.0 | 37.5 | 51.7 | 454 | 41.2 | 27.5 | 5.1 |
| Pirojpur | 12.2 | 37.8 | 48.6 | 813 | 43.9 | 27.5 | 5.2 | 15.1 | 35.9 | 64.3 | 849 | 43.3 | 28.9 | 4.8 |
| Bandarban | 23.7 | 52.7 | 23.8 | 52 | 39.1 | 27.9 | 5.0 | 31.1 | 44.0 | 31.7 | 67 | 42.5 | 32.4 | 5.0 |
| Brahmanbaria | 12.1 | 41.9 | 26.6 | 1112 | 42.1 | 23.8 | 6.0 | 14.0 | 36.0 | 39.5 | 1245 | 46.0 | 30.4 | 5.6 |
| Chandpur | 9.3 | 37.7 | 37.8 | 1192 | 45.3 | 26.8 | 5.7 | 13.8 | 33.7 | 50.3 | 1333 | 47.9 | 32.3 | 5.2 |
| Chattogram | 45.5 | 43.0 | 43.2 | 1002 | 49.5 | 33.1 | 5.8 | 51.1 | 38.7 | 55.6 | 1252 | 52.5 | 37.3 | 5.3 |
| Cumilla | 9.0 | 39.7 | 33.1 | 1307 | 43.0 | 25.5 | 5.9 | 11.6 | 35.5 | 46.0 | 1490 | 47.7 | 32.5 | 5.6 |
| Cox's Bazar | 13.6 | 44.6 | 21.9 | 569 | 46.5 | 30.2 | 6.5 | 15.3 | 36.4 | 30.2 | 712 | 51.4 | 37.5 | 6.0 |
| Feni | 8.7 | 36.8 | 40.7 | 1182 | 47.9 | 28.7 | 5.9 | 13.7 | 32.5 | 54.3 | 1337 | 52.2 | 34.2 | 5.6 |
| Khagrachari | 32.0 | 47.1 | 26.3 | 127 | 38.2 | 26.7 | 4.8 | 32.5 | 40.9 | 41.8 | 195 | 40.1 | 29.8 | 4.8 |
| Lakshmipur | 14.1 | 39.3 | 34.3 | 901 | 43.1 | 25.5 | 5.5 | 15.1 | 34.6 | 42.9 | 1023 | 47.3 | 31.0 | 5.2 |
| Noakhali | 10.3 | 36.8 | 37.1 | 616 | 46.1 | 26.6 | 5.8 | 13.7 | 32.5 | 51.7 | 716 | 49.9 | 33.0 | 5.6 |
| Rangamati | 36.4 | 49.5 | 36.5 | 66 | 40.9 | 31.0 | 5.3 | 33.5 | 46.6 | 43.6 | 83 | 42.6 | 33.4 | 4.9 |
| Dhaka | 88.1 | 49.8 | 53.9 | 3989 | 46.9 | 32.2 | 5.6 | 91.6 | 48.2 | 64.8 | 5814 | 48.5 | 34.9 | 4.7 |
| Faridpur | 10.3 | 41.6 | 27.8 | 726 | 42.5 | 24.8 | 5.4 | 13.0 | 38.0 | 40.9 | 847 | 43.3 | 28.6 | 5.0 |
| Gazipur | 39.0 | 44.0 | 36.6 | 932 | 39.8 | 23.8 | 5.1 | 45.7 | 43.9 | 56.4 | 1129 | 41.6 | 28.0 | 4.5 |
| Gopalganj | 7.0 | 38.0 | 38.2 | 712 | 46.1 | 28.4 | 5.5 | 9.7 | 36.6 | 51.3 | 782 | 46.2 | 30.7 | 5.3 |
| Jamalpur | 11.3 | 46.9 | 21.5 | 922 | 37.9 | 20.6 | 4.9 | 15.7 | 39.9 | 31.8 | 1037 | 38.4 | 23.8 | 4.4 |
| Kishoreganj | 12.3 | 44.9 | 23.3 | 858 | 40.3 | 22.2 | 5.5 | 13.8 | 40.8 | 38.2 | 965 | 41.8 | 26.2 | 4.9 |
| Madaripur | 8.0 | 42.8 | 32.6 | 934 | 42.5 | 25.9 | 5.4 | 12.2 | 25.3 | 42.1 | 1001 | 44.8 | 30.5 | 5.0 |
| Manikganj | 7.8 | 40.4 | 26.9 | 853 | 41.1 | 23.1 | 5.2 | 7.4 | 38.3 | 41.0 | 932 | 40.2 | 25.1 | 4.6 |
| Munshiganj | 9.2 | 40.0 | 35.8 | 1244 | 47.1 | 31.4 | 5.8 | 11.5 | 34.9 | 51.6 | 1355 | 48.3 | 33.5 | 5.2 |
| Mymensingh | 12.9 | 44.6 | 25.5 | 907 | 38.5 | 21.7 | 5.2 | 14.7 | 41.2 | 39.1 | 1029 | 39.8 | 26.0 | 4.7 |
| Narayanganj | 51.1 | 46.4 | 39.5 | 2312 | 42.7 | 28.1 | 5.6 | 56.2 | 42.3 | 51.8 | 3106 | 45.2 | 31.4 | 4.8 |
| Narsingdi | 16.1 | 43.8 | 29.6 | 1447 | 39.2 | 22.9 | 5.4 | 18.4 | 39.0 | 42.9 | 1662 | 42.5 | 28.5 | 4.9 |
| Netrokona | 7.8 | 45.6 | 26.0 | 616 | 43.3 | 24.3 | 5.3 | 9.4 | 47.7 | 34.9 | 708 | 43.2 | 27.1 | 4.9 |
| Rajbari | 10.3 | 42.3 | 26.4 | 746 | 42.2 | 24.3 | 5.7 | 12.5 | 38.1 | 39.8 | 851 | 42.8 | 28.0 | 5.0 |
| Shariatpur | 7.2 | 40.5 | 24.4 | 807 | 43.2 | 26.0 | 5.4 | 10.6 | 36.7 | 39.0 | 916 | 45.4 | 31.6 | 5.1 |
| Sherpur | 9.5 | 46.4 | 19.5 | 835 | 35.2 | 18.9 | 4.9 | 10.6 | 41.4 | 31.9 | 938 | 37.3 | 23.6 | 4.2 |
| Tangail | 9.4 | 41.5 | 29.4 | 875 | 39.9 | 22.8 | 5.3 | 13.3 | 38.3 | 40.5 | 964 | 40.3 | 25.5 | 4.6 |
| Bagerhat | 13.2 | 39.2 | 44.3 | 361 | 42.3 | 25.7 | 5.2 | 13.3 | 38.4 | 58.7 | 391 | 41.9 | 27.2 | 4.8 |
| Chuadanga | 26.0 | 42.1 | 25.2 | 697 | 39.8 | 22.9 | 5.5 | 27.3 | 39.6 | 40.9 | 856 | 37.7 | 23.4 | 4.5 |
| Jashore | 13.4 | 41.8 | 33.4 | 821 | 40.7 | 23.9 | 5.5 | 16.2 | 38.8 | 51.3 | 963 | 40.4 | 25.7 | 4.7 |
| Jhenaidah | 12.8 | 42.0 | 25.9 | 694 | 40.7 | 23.7 | 5.7 | 14.6 | 39.1 | 44.7 | 805 | 40.1 | 25.2 | 4.7 |
| Khulna | 50.1 | 42.6 | 43.9 | 458 | 41.9 | 25.9 | 5.4 | 54.0 | 38.6 | 57.8 | 541 | 42.9 | 28.4 | 4.8 |
| Kushtia | 11.0 | 42.7 | 25.8 | 927 | 41.8 | 24.1 | 5.5 | 12.3 | 37.9 | 40.4 | 1087 | 40.7 | 25.0 | 4.6 |
| Magura | 7.2 | 39.0 | 28.2 | 690 | 43.2 | 25.3 | 5.8 | 12.8 | 37.7 | 44.7 | 786 | 42.1 | 27.2 | 5.0 |
| Meherpur | 9.3 | 42.5 | 23.1 | 687 | 38.9 | 22.4 | 5.3 | 11.5 | 40.4 | 37.8 | 826 | 37.1 | 22.3 | 4.2 |
| Narail | 10.2 | 39.0 | 35.2 | 663 | 45.3 | 26.5 | 5.6 | 12.3 | 36.6 | 48.6 | 706 | 43.5 | 27.4 | 5.0 |
| Satkhira | 8.3 | 41.9 | 30.5 | 414 | 38.3 | 20.8 | 5.5 | 9.2 | 37.8 | 45.5 | 483 | 40.0 | 25.2 | 4.8 |
| Bogura | 11.1 | 44.0 | 28.4 | 914 | 37.7 | 21.8 | 5.2 | 12.9 | 39.5 | 42.9 | 1032 | 38.6 | 24.6 | 3.1 |
| Joypurhat | 10.2 | 45.0 | 30.2 | 793 | 36.3 | 20.3 | 5.1 | 14.3 | 40.0 | 49.6 | 877 | 36.1 | 22.0 | 4.1 |
| Naogaon | 7.9 | 44.4 | 28.4 | 625 | 36.9 | 20.0 | 5.4 | 9.3 | 40.1 | 44.4 | 696 | 37.3 | 23.0 | 4.4 |
| Natore | 11.5 | 42.5 | 26.9 | 732 | 40.7 | 23.8 | 5.5 | 12.6 | 39.7 | 41.6 | 802 | 39.6 | 24.9 | 4.5 |
| Chapai Nawabganj | 18.6 | 40.6 | 23.8 | 688 | 39.1 | 24.3 | 6.0 | 18.9 | 36.2 | 35.9 | 837 | 40.0 | 27.3 | 5.2 |
| Pabna | 17.6 | 44.0 | 26.8 | 810 | 42.6 | 25.7 | 5.9 | 20.6 | 38.6 | 42.4 | 917 | 42.7 | 28.9 | 4.9 |
| Rajshahi | 30.6 | 41.9 | 30.6 | 784 | 41.5 | 25.1 | 5.3 | 36.9 | 37.9 | 47.5 | 950 | 41.4 | 26.4 | 4.6 |
| Sirajganj | 11.2 | 44.4 | 27.0 | 906 | 40.4 | 24.0 | 5.6 | 11.9 | 38.9 | 40.6 | 1078 | 41.8 | 28.2 | 4.8 |
| Dinajpur | 12.7 | 44.1 | 29.8 | 657 | 38.2 | 21.0 | 5.3 | 14.0 | 39.3 | 45.7 | 769 | 39.4 | 25.3 | 4.6 |
| Gaibandha | 7.1 | 42.9 | 24.3 | 894 | 37.3 | 19.3 | 5.2 | 9.1 | 38.7 | 35.7 | 981 | 38.2 | 23.7 | 4.3 |
| Kurigram | 14.1 | 43.6 | 22.3 | 698 | 38.8 | 18.4 | 5.4 | 15.5 | 65.8 | 33.5 | 781 | 39.2 | 23.2 | 4.5 |
| Lalmonirhat | 11.0 | 46.2 | 23.8 | 767 | 38.9 | 19.8 | 5.2 | 12.7 | 39.4 | 42.3 | 894 | 39.1 | 24.2 | 4.5 |
| Nilphamari | 13.7 | 45.4 | 25.3 | 822 | 36.5 | 19.6 | 5.2 | 15.0 | 39.2 | 38.8 | 995 | 38.8 | 25.0 | 4.7 |
| Panchagarh | 8.2 | 44.5 | 30.9 | 507 | 39.5 | 21.4 | 5.1 | 8.6 | 40.1 | 43.9 | 595 | 41.3 | 27.1 | 4.7 |
| Rangpur | 16.7 | 44.3 | 26.7 | 936 | 38.6 | 20.9 | 5.1 | 18.0 | 38.8 | 41.9 | 1074 | 39.9 | 25.6 | 4.4 |
| Thakurgaon | 9.3 | 44.7 | 27.3 | 559 | 39.9 | 23.3 | 5.2 | 9.7 | 39.9 | 41.8 | 671 | 40.4 | 28.5 | 4.7 |
| Habiganj | 7.8 | 44.8 | 24.5 | 579 | 41.6 | 25.0 | 5.7 | 10.9 | 40.2 | 37.7 | 667 | 45.8 | 31.4 | 5.5 |
| Maulvibazar | 7.9 | 46.3 | 30.8 | 492 | 45.1 | 28.8 | 5.7 | 9.0 | 39.4 | 42.1 | 576 | 48.4 | 33.8 | 5.5 |
| Sunamganj | 7.1 | 45.4 | 22.3 | 466 | 45.2 | 27.8 | 6.1 | 10.8 | 41.2 | 34.4 | 549 | 47.5 | 32.2 | 5.8 |
| Sylhet | 15.4 | 42.5 | 33.8 | 617 | 48.9 | 31.8 | 6.3 | 16.9 | 37.0 | 45.6 | 732 | 52.6 | 37.3 | 6.0 |

Note: UR: Urbanization rate, AR: Activity rate; LR: Literacy rate; PD: Population density per square mile; NMM: Percentage of never-married male; NMF: percentage of never-married female; HS: Average household size.
