# Supplementary material for: Dynamics of internal migration in Bangladesh: Trends, patterns, determinants, and causes
Source: PLoS One. 2022 Feb 14;17(2):e0263878. doi: 10.1371/journal.pone.0263878 (PMC8843202; doi:10.1371/journal.pone.0263878)
Supplement: S3 Table — (DOCX) [file pone.0263878.s003.docx]

**S3 Table.** Lifetime internal migration by districts of Bangladesh, 1991 and 2001

| **District** | **Internal Migration (%), 1991** | | | | | | | **Internal Migration (%), 2001** | | | | | | |
| --- | --- | --- | --- | --- | --- | --- | --- | --- | --- | --- | --- | --- | --- | --- |
|  | **Within district** | | | **Outside district** | | | **Total** | **Within district** | | | **Outside district** | | | **Total** |
|  | **UtR** | **RtR** | **RtU** | **RtU** | **UtR** | **UtU** |  | **UtR** | **RtR** | **RtU** | **RtU** | **UtR** | **UtU** |  |
| Barguna | 0.0 | 3.2 | 0.7 | 1.5 | 0.3 | 0.6 | 0.4 | 0.9 | 1.2 | 0.4 |  | 0.2 | 2.7 | 3.7 |
| Barishal | 0.1 | 66.2 | 16.1 | 145.3 | 0.4 | 8.9 | 0.9 | 0.4 | 1.7 | 0.3 |  | 0.3 | 6.6 | 3.0 |
| Bhola | 0.1 | 90.6 | 40.9 | 77.8 | 0.1 | 40.9 | 0.4 | 0.3 | 1.4 | 0.1 |  | 0.0 | 2.2 | 1.7 |
| Jhalokathi | 0.0 | 19.8 | 15.8 | 11.6 | 0.1 | 17.0 | 0.3 | 1.0 | 0.8 | 0.5 |  | 0.3 | 1.2 | 4.8 |
| Patuakhali | 0.1 | 104.3 | 27.5 | 70.5 | 0.0 | 30.8 | 0.5 | 1.0 | 0.9 | 0.1 |  | 0.2 | 1.2 | 0.8 |
| Pirojpur | 0.0 | 143.3 | 25.0 | 44.2 | 0.2 | 17.0 | 0.4 | 0.8 | 0.5 | 0.3 |  | 0.2 | 2.6 | 3.0 |
| Bandarban | 0.0 | 89.7 | 13.1 | 8.6 | 0.2 | 3.4 | 0.6 |  | 0.0 | 0.0 |  |  | 0.0 | 19.7 |
| Brahmanbaria | 0.0 | 13.2 | 4.5 | 5.3 | 0.3 | 2.6 | 2.4 | 0.1 | 0.4 | 0.3 | 0.4 | 0.5 | 1.0 | 1.6 |
| Chandpur | 0.0 | 38.7 | 44.7 | 54.2 | 0.1 | 8.3 | 1.0 | 0.2 | 0.6 | 0.5 | 0.0 | 0.5 | 2.8 | 4.8 |
| Chattogram | 0.5 | 4.3 | 49.1 | 2.1 | 0.2 | 7.7 | 12.3 | 0.2 | 0.7 | 1.0 | 0.0 | 0.3 | 0.5 | 12.9 |
| Cumilla | 0.1 | 120.2 | 30.5 | 141.2 | 0.2 | 7.7 | 1.3 | 0.4 | 0.6 | 0.2 | 0.1 | 0.4 | 2.1 | 1.9 |
| Cox's Bazar | 0.1 | 89.7 | 26.8 | 8.2 | 0.0 | 51.2 | 0.6 | 0.8 | 0.7 | 0.0 | 0.2 | 0.4 | 0.3 | 2.6 |
| Feni | 0.0 | 112.6 | 27.6 | 72.4 | 0.2 | 8.6 | 0.3 | 0.4 | 0.5 | 0.1 |  | 0.0 | 0.9 | 2.8 |
| Khagrachari | 0.1 | 14.7 | 25.9 | 5.3 | 0.0 | 88.8 | 0.9 | 1.5 | 0.6 | 0.1 |  | 0.1 | 0.5 | 20.1 |
| Lakshmipur | 0.0 | 73.6 | 96.3 | 21.0 | 0.1 | 1.2 | 0.2 | 3.5 | 0.7 | 0.1 | 0.6 | 0.3 | 0.8 | 1.9 |
| Noakhali | 0.1 | 96.4 | 53.7 | 104.5 | 0.3 | 24.3 | 1.2 | 0.1 | 0.6 | 0.1 |  | 0.1 | 1.9 | 1.9 |
| Rangamati | 0.0 | 19.3 | 25.1 | 5.8 | 0.2 | 11.1 | 1.1 | 0.4 | 1.7 | 0.1 |  | 0.1 | 0.1 | 18.7 |
| Dhaka | 0.1 | 4.6 | 25.0 | 2.7 | 5.0 | 0.9 | 23.2 | 0.1 | 1.3 | 7.0 | 0.0 | 1.3 | 1.7 | 49.0 |
| Faridpur | 0.1 | 153.1 | 16.7 | 306.7 | 0.5 | 42.7 | 0.8 | 0.3 | 1.3 | 0.3 |  | 0.6 | 3.8 | 4.1 |
| Gazipur | 0.0 | 6.1 | 40.7 | 2.6 | 0.0 | 0.7 | 2.8 | 0.4 | 1.1 | 2.1 |  | 0.1 | 0.8 | 27.6 |
| Gopalganj | 0.0 | 112.0 | 28.7 | 125.8 | 0.1 | 17.4 | 0.4 | 1.5 | 0.4 | 0.2 |  | 0.3 | 1.2 | 5.7 |
| Jamalpur | 0.0 | 38.8 | 31.1 | 21.5 | 0.1 | 2.7 | 0.7 | 0.5 | 0.7 | 0.2 |  | 0.1 | 1.2 | 2.7 |
| Kishoreganj | 0.0 | 53.5 | 90.9 | 25.7 | 0.1 | 2.4 | 1.0 | 1.7 | 0.6 | 0.1 |  | 0.2 | 2.1 | 3.4 |
| Madaripur | 0.1 | 82.9 | 41.8 | 58.2 | 0.1 | 72.8 | 0.6 | 1.3 | 1.5 | 0.1 |  | 0.1 | 1.7 | 3.7 |
| Manikganj | 0.0 | 166.0 | 18.3 | 59.2 | 0.2 | 10.6 | 0.4 | 0.5 | 1.0 | 0.1 | 0.1 | 0.4 | 1.3 | 13.0 |
| Munshiganj | 0.0 | 44.2 | 47.5 | 177.0 | 0.1 | 6.6 | 0.5 | 0.2 | 0.5 | 0.0 | 0.1 | 0.1 | 3.0 | 6.1 |
| Mymensingh | 0.0 | 113.7 | 26.1 | 37.1 | 0.1 | 2.8 | 1.3 | 2.0 | 0.7 | 0.2 | 0.2 | 0.4 | 2.2 | 3.3 |
| Narayanganj | 0.2 | 3.3 | 30.1 | 2.5 | 0.3 | 10.2 | 2.2 | 0.2 | 0.9 | 2.5 | 0.1 | 0.1 | 1.3 | 27.6 |
| Narsingdi | 0.1 | 41.9 | 22.5 | 16.7 | 0.1 | 28.6 | 1.1 | 0.7 | 1.0 | 0.1 |  | 0.2 | 1.1 | 5.4 |
| Netrokona | 0.0 | 67.7 | 38.0 | 17.4 | 0.0 | 5.4 | 0.6 | 1.1 | 0.5 |  |  | 0.1 | 0.8 | 3.2 |
| Rajbari | 0.0 | 16.4 | 40.2 | 6.6 | 0.1 | 2.4 | 0.8 | 0.7 | 1.1 | 0.1 |  | 0.0 | 2.7 | 7.6 |
| Shariatpur | 0.1 | 85.4 | 11.9 | 67.6 | 0.1 | 98.6 | 0.5 | 1.0 | 1.6 | 0.1 |  | 0.0 | 2.7 | 4.2 |
| Sherpur | 0.1 | 24.9 | 15.3 | 12.8 | 0.0 | 43.3 | 0.7 | 0.8 | 1.6 | 0.0 |  | 0.1 | 1.9 | 3.7 |
| Tangail | 0.0 | 53.5 | 25.5 | 29.9 | 0.1 | 6.0 | 0.8 | 0.2 | 0.9 | 0.2 | 0.1 | 0.2 | 0.9 | 2.1 |
| Bagerhat | 0.0 | 27.3 | 47.0 | 27.2 | 0.3 | 1.2 | 0.8 | 1.2 | 0.9 | 0.3 | 0.1 | 0.3 | 1.9 | 5.8 |
| Chuadanga | 0.1 | 33.2 | 59.2 | 3.2 | 0.1 | 22.1 | 1.4 | 0.2 | 0.6 | 0.3 |  | 1.5 | 1.0 | 7.0 |
| Jashore | 0.1 | 45.8 | 14.7 | 24.2 | 0.2 | 10.8 | 1.9 | 0.8 | 0.6 | 0.2 | 0.1 | 0.2 | 0.7 | 8.5 |
| Jhenaidah | 0.0 | 26.7 | 30.6 | 3.0 | 0.2 | 1.3 | 1.5 | 0.4 | 0.5 | 0.6 |  | 0.2 | 0.3 | 7.0 |
| Khulna | 0.0 | 9.0 | 42.0 | 4.4 | 1.1 | 1.0 | 5.8 | 0.1 | 0.8 | 1.2 | 0.0 | 0.3 | 1.6 | 20.9 |
| Kushtia | 0.1 | 62.7 | 20.0 | 20.5 | 0.2 | 13.0 | 1.1 | 0.8 | 0.6 | 0.2 |  | 0.2 | 0.9 | 4.4 |
| Magura | 0.0 | 60.4 | 33.2 | 18.0 | 0.4 | 6.7 | 0.6 | 0.4 | 0.5 | 0.4 |  | 0.4 | 0.7 | 8.8 |
| Meherpur | 0.0 | 68.6 | 15.7 | 14.7 | 0.1 | 13.1 | 1.1 | 5.3 | 0.4 | 0.1 |  | 0.1 | 0.1 | 3.9 |
| Narail | 0.0 | 42.1 | 34.4 | 25.2 | 0.1 | 22.1 | 0.6 | 0.5 | 1.0 | 0.1 | 0.3 | 0.1 | 0.9 | 9.0 |
| Satkhira | 0.0 | 28.9 | 41.0 | 8.1 | 0.0 | 2.9 | 0.9 | 0.3 | 1.5 | 0.2 | 0.4 | 0.2 | 0.5 | 4.0 |
| Bogura | 0.0 | 45.4 | 32.2 | 15.9 | 0.1 | 4.2 | 1.2 | 1.8 | 0.5 | 0.2 | 0.2 | 0.6 | 0.4 | 4.7 |
| Joypurhat | 0.0 | 48.9 | 17.6 | 14.4 | 0.1 | 24.3 | 0.6 |  | 1.0 | 0.2 |  | 0.0 | 0.3 | 7.3 |
| Naogaon | 0.0 | 0.0 | 0.0 | 0.0 | 0.0 | 0.0 | 1.3 | 1.9 | 0.6 | 0.1 | 0.1 | 0.0 | 0.3 | 3.1 |
| Natore | 0.0 | 20.8 | 25.2 | 7.1 | 0.1 | 2.2 | 1.3 | 0.3 | 0.4 | 0.1 |  | 0.1 | 0.3 | 5.4 |
| Chapai Nawabganj | 0.0 | 12.6 | 24.8 | 3.6 | 0.1 | 1.4 | 0.7 | 1.2 | 4.7 |  | 1.2 | 0.1 | 0.3 | 34.8 |
| Pabna | 0.1 | 27.3 | 46.6 | 15.8 | 0.2 | 6.0 | 1.2 | 0.3 | 0.5 | 0.1 |  | 0.2 | 1.2 | 2.6 |
| Rajshahi | 0.0 | 12.9 | 57.3 | 5.5 | 0.4 | 0.6 | 3.5 | 0.4 | 0.6 | 0.6 | 0.0 | 0.3 | 0.7 | 4.6 |
| Sirajganj | 0.0 | 19.7 | 30.8 | 13.2 | 0.0 | 3.7 | 0.7 | 0.7 | 0.6 | 0.0 |  | 0.0 | 0.8 | 1.6 |
| Dinajpur | 0.1 | 19.3 | 12.7 | 8.5 | 0.1 | 11.3 | 1.6 | 0.5 | 0.6 | 0.1 | 0.1 | 0.1 | 0.6 | 5.5 |
| Gaibandha | 0.1 | 166.0 | 22.4 | 45.6 | 0.1 | 40.7 | 0.5 | 1.6 | 0.5 |  |  | 0.1 | 0.4 | 2.1 |
| Kurigram | 0.0 | 38.1 | 12.4 | 19.5 | 0.1 | 5.4 | 0.4 | 2.6 | 1.2 | 0.0 |  | 0.2 | 0.5 | 1.6 |
| Lalmonirhat | 0.0 | 18.8 | 17.6 | 9.7 | 0.1 | 3.3 | 0.8 | 1.5 | 1.1 | 0.0 |  | 0.2 | 0.8 | 4.2 |
| Nilphamari | 0.0 | 7.7 | 16.9 | 2.4 | 0.0 | 5.7 | 0.8 | 0.2 | 0.4 | 0.0 |  | 0.1 | 0.2 | 3.7 |
| Panchagarh | 0.0 | 16.4 | 12.9 | 8.0 | 0.0 | 8.0 | 0.8 | 0.5 | 0.3 | 0.2 |  | 0.2 | 0.4 | 6.7 |
| Rangpur | 0.3 | 31.3 | 22.6 | 11.4 | 0.1 | 25.4 | 2.2 | 1.0 | 0.6 | 0.1 | 0.2 | 0.1 | 0.9 | 4.5 |
| Thakurgaon | 0.0 | 23.2 | 22.2 | 3.3 | 0.1 | 3.0 | 0.8 | 1.6 | 0.5 | 0.0 |  |  | 0.5 | 4.9 |
| Habiganj | 0.0 | 69.2 | 40.6 | 18.8 | 0.0 | 30.4 | 1.0 | 4.6 | 1.1 | 0.1 |  | 0.1 | 0.2 | 4.0 |
| Maulvibazar | 0.0 | 112.9 | 23.2 | 5.3 | 0.0 | 13.2 | 0.7 | 0.6 | 0.2 | 0.0 | 0.4 |  | 0.1 | 4.1 |
| Sunamganj | 0.0 | 65.9 | 28.3 | 13.9 | 0.0 | 22.5 | 1.0 | 3.1 | 0.4 | 0.1 |  | 0.1 | 0.2 | 3.4 |
| Sylhet | 0.1 | 35.7 | 17.3 | 20.9 | 0.2 | 34.3 | 1.1 | 20.8 | 0.8 | 0.1 |  | 0.4 | 0.5 | 8.6 |

Note: RtR: Rural to rural; RtU: Rural to urban; UtR: Urban to rural; UtU: Urban to urban.
